# Lysyl oxidase drives tumour progression by trapping EGF receptors at the cell surface

**DOI:** 10.1038/ncomms14909

**Published:** 2017-04-18

**Authors:** HaoRan Tang, Leo Leung, Grazia Saturno, Amaya Viros, Duncan Smith, Gianpiero Di Leva, Eamonn Morrison, Dan Niculescu-Duvaz, Filipa Lopes, Louise Johnson, Nathalie Dhomen, Caroline Springer, Richard Marais

**Affiliations:** 1 Molecular Oncology Group, Cancer Research UK Manchester Institute, University of Manchester, Manchester M20 4BX, UK; 2 Gene and Oncogene Targeting Team, CRUK Cancer Therapeutics Unit, The Institute of Cancer Research, London SM2 5NG, UK; 3 Biological Mass Spectrometry Unit, Cancer Research UK Manchester Institute, University of Manchester, Manchester M20 4BX, UK

## Abstract

Lysyl oxidase (LOX) remodels the tumour microenvironment by cross-linking the extracellular matrix. LOX overexpression is associated with poor cancer outcomes. Here, we find that LOX regulates the epidermal growth factor receptor (EGFR) to drive tumour progression. We show that LOX regulates EGFR by suppressing TGFβ1 signalling through the secreted protease HTRA1. This increases the expression of Matrilin2 (MATN2), an EGF-like domain-containing protein that traps EGFR at the cell surface to facilitate its activation by EGF. We describe a pharmacological inhibitor of LOX, CCT365623, which disrupts EGFR cell surface retention and delays the growth of primary and metastatic tumour cells *in vivo*. Thus, we show that LOX regulates EGFR cell surface retention to drive tumour progression, and we validate the therapeutic potential of inhibiting this pathway with the small molecule inhibitor CCT365623.

## Abstract (web summary)

Lysyl oxidase is able to remodel the extracellular matrix and its expression correlates with poor prognosis. Here the authors show that this protein modulates trapping of the epidermal growth factor receptor at the cell surface, causing persistent signalling activation and tumour progression.

Lysyl oxidase (LOX) is a copper and quinone-cofactor containing amine oxidase that is secreted as an inactive pro-enzyme, and is then cleaved to a mature active form by extracellular metalloproteases such as BMP-1 (ref. 1). LOX catalyses the formation of covalent bonds in collagens and elastin and thereby increases the stiffness of the extracellular matrix (ECM), increasing the tensile strength of extracellular fibres^2^,^3^. Importantly, LOX is also implicated in cancer, because high LOX expression correlates to poor outcome in colon, breast, pancreas, prostate and lung cancers^4^,^5^,^6^,^7^,^8^. Secreted LOX is reported to cross-link the ECM at distal sites to create pre-metastatic niches to which bone marrow-derived and tumour cells are recruited, facilitating the metastatic spread of cancer^9^. Moreover, within the tumour LOX-mediated ECM stiffening is reported to drive integrin activation and tumour initiation in a mouse model of breast cancer^10^, and also to drive the growth of the primary tumours in colorectal cancer^11^.

Thus, by modulating tissue stiffness, LOX promotes tumour progression, but intriguingly, when myofibroblasts are depleted in a mouse pancreatic cancer model, collagen content and tissue stiffness are reduced, but disease progression is accelerated^12^. Notably, LOX expression remains strong in this model suggesting that LOX functions beyond regulating matrix stiffness, and a recent study demonstrates that secreted LOX induces bone pre-metastatic lesion formation by regulating NFATc1 driven osteoclastogenesis^13^. Extracellular LOX is also reported to regulate signalling by the platelet-derived growth factor receptor (PDGFR)^14^ and transforming growth factor β1 (TGFβ1)^15^,^16^. PDGFR regulation by LOX is implicated in angiogenesis^17^, but how LOX controls intracellular signalling to drive tumour progression is still poorly understood.

In this study, we examined how LOX regulates intracellular signalling. Using an unbiased approach, we show that LOX regulates the EGF receptor (EGFR). We also show that LOX inhibits TGFβ1 through the serine protease HTRA1, and that this increases expression of the secreted protein MATN2. MATN2 traps EGFR at the cell surface for enhanced activation by EGF, driving tumour progression. Moreover *in vivo* inhibition of LOX by our inhibitor CCT365623 disrupts this signalling axis and reduces tumour progression.

## Results

### LOX regulates the EGFR

**Figure 1:**
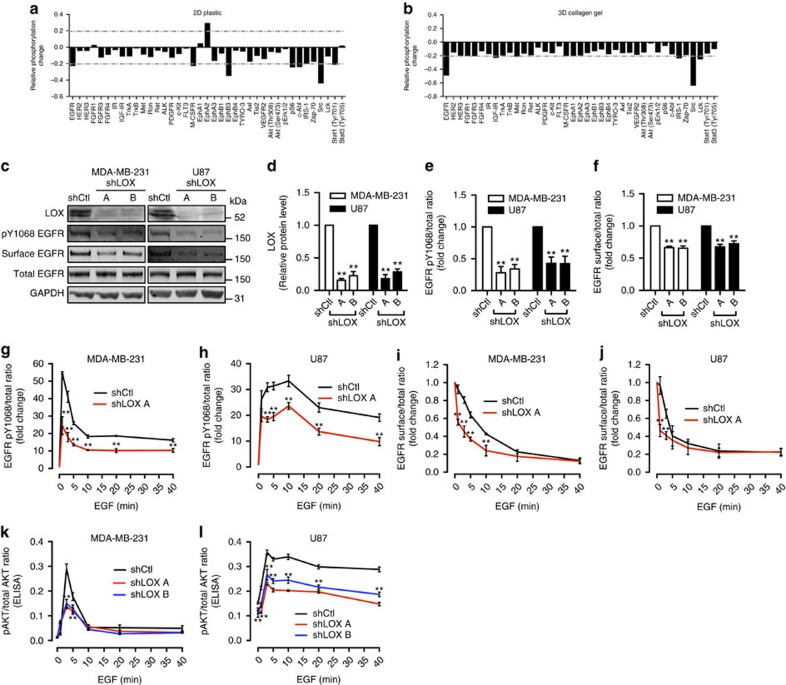
LOX regulates EGFR activation and cell surface retention. (**a**) Protein phosphorylation changes in LOX-depleted MDA-MB-231 cells on 2D plastic and (**b**) in 3D collagen gels as shown by a RTK antibody array (±20% arbitrary cut-off). (**c**) Western blots of LOX, pY1068 EGFR, surface EGFR, total EGFR and GAPDH in control (shCtl) or LOX-depleted (shLOX A,B) MDA-MB-231 and U87 cells. (**d**) Quantification of LOX protein expression, (**e**) EGFR activation and (**f**) surface EGFR level in MDA-MB-231 and U87 cells from experiments in **c**. All data are represented as mean±s.d. from three independent experiments. **P<0.01, Student's *t*-test. (**g**,**h**) Levels of EGFR activation and (**i**,**j**) surface EGFR in MDA-MB-231 or U87 control (shCtl) or LOX-depleted (shLOX A) cells following EGF stimulation for indicated time. (**k**,**l**) AKT activation in MDA-MB-231 or U87 control (shCtl) or LOX-depleted (shLOX A,B) cells following EGF stimulation for indicated time. All data in **g**–**l** are represented as mean±s.d. from three independent experiments at each indicated time point. **P<0.01, Student's *t*-test.

To discover unidentified functions of LOX, we examined how its depletion by short-hairpin RNA (shRNA) affects cell signalling using an antibody array to measure receptor tyrosine kinase (RTK) phosphorylation in MDA-MB-231 cells. We found that when LOX was depleted in MDA-MB-231 breast cancer cells grown in standard plastic (2D) culture conditions, phosphorylation of several RTKs including the EGFR was reduced (Fig. 1a). LOX cross-links collagen and drives cell invasion and metastasis, so we performed this assay on cells grown within collagen gels (3D) and observed that the strongest effects of LOX depletion are on EGFR and SRC phosphorylation (Fig. 1b). Although LOX has previously been linked to SRC^8^, its role in EGFR signalling has not been reported, but like LOX, EGFR is implicated in both tumour cell growth and metastasis. For example, in breast cancer paracrine EGF signalling by tumour-associated macrophages promoted breast cancer cell invasion^18^ and the EGFR ligand heparin-binding EGF-like growth factor (HBEGF) was implicated in brain metastasis^19^. Since our results show the link from LOX to EGFR, we focused on their interaction.

By western blot, we confirm that in standard 2D tissue culture conditions LOX depletion by shRNA causes reduced phosphorylation of EGFR in MDA-MB-231 and U87 cells (Fig. 1c–e). Critically, this occurs without impacting the levels of total EGFR in the cells (Fig. 1c), so we examined whether LOX depletion affects surface retention of EGFR by biotinylating the intact cells and capturing the biotinylated surface proteins on streptavidin beads for western blot analysis. Notably, LOX depletion caused a substantial reduction in the levels of EGFR at the cell surface in MDA-MB-231 and U87 cells (Fig. 1c,f). Next, we examined EGFR activation by EGF and found that LOX depletion suppressed EGF stimulated EGFR phosphorylation in MDA-MB-231 and U87 cells (Fig. 1g,h; Supplementary Fig. 1a,b). Moreover, when LOX was depleted, EGF stimulated more rapid loss of EGFR from the cell surface (Fig. 1i,j; Supplementary Fig. 1a,b). Note that LOX depletion also reduced EGF-stimulated phosphorylation of the downstream signalling molecule AKT (Fig. 1k,l). Note that these effects on EGF-stimulated cell signalling were sustained, because EGFR phosphorylation and surface retention, and also AKT phosphorylation were suppressed for up to 24 h (Supplementary Fig. 1c–g). Altogether, these results show that LOX modulates EGFR retention at the cell surface and thereby regulates the fidelity of signalling by this RTK.

**Figure 2:**
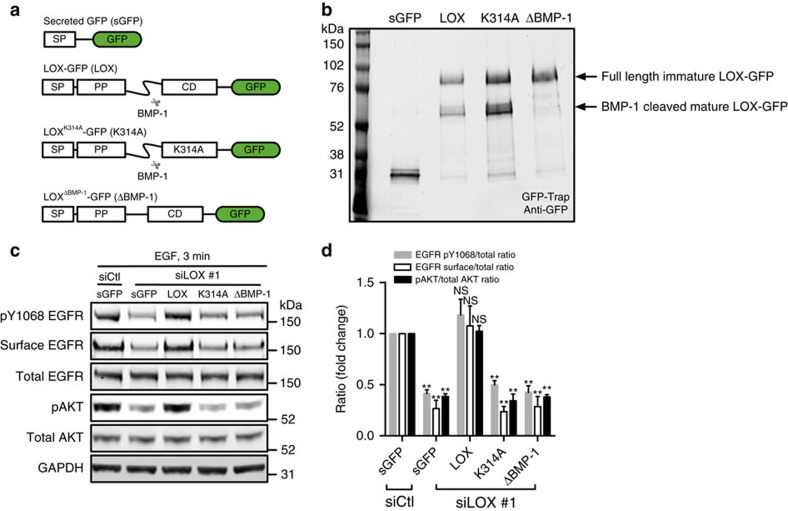
LOX activity controls EGFR cell surface retention and activation. (**a**) Schematic illustrating the secreted green fluorescent protein (sGFP, control), and various LOX-GFP expression constructs used in this study. SP, signal peptide; PP, pro-peptide; CD, catalytic domain; LOX, wild-type enzyme; K314A, catalytically inactive LOX mutant; ΔBMP-1; BMP-1 cleavage site-deleted LOX mutant. (**b**) Western blot showing immunoprecipitated sGFP and indicated LOX-GFP fusion proteins from MDA-MB-231 cell culture medium. (**c**) Western blots for pY1068 EGFR, surface EGFR, total EGFR, pAKT, total AKT and GAPDH in EGF-stimulated (3 min) control (siCtl) or LOX-depleted (siLOX #1) MDA-MB-231 cells expressing sGFP, and indicated LOX-GFP fusion proteins. (**d**) Quantification of experiments in **c**. Data are represented as mean±s.d. from four independent experiments. **P<0.01, Student's *t*-test.

To establish if the LOX catalytic activity is responsible for regulating EGFR function, we transiently deplete endogenous LOX from MDA-MB-231 cells and test if LOX re-expression restores EGFR function (Supplementary Fig. 2a–c). For these studies, we use LOX fused to GFP, testing wild-type LOX (LOX-GFP), a catalytically inactive mutant of LOX (LOX^K314A^-GFP), a LOX mutant that cannot be cleaved to the mature form by BMP-1 (LOX^ΔBMP-1^-GFP), and the LOX signal peptide fused directly to GFP (sGFP; Fig. 2a). We confirm that all four proteins are secreted and that LOX^ΔBMP-1^-GFP is not cleaved to the mature active form (Fig. 2b). We confirm that depletion of endogenous LOX reduces surface levels of EGFR, and also suppresses EGFR and AKT phosphorylation (Fig. 2c,d). Critically, EGFR surface retention, and EGFR and AKT phosphorylation are restored by LOX-GFP, but not by sGFP, LOX^K314A^-GFP or LOX^ΔBMP-1^-GFP (Fig. 2c,d). Thus, we conclude that EGFR cell surface retention and signalling require secretion of mature and active LOX.

### LOX regulates MATN2 expression through TGFβ1 signalling

**Figure 3:**
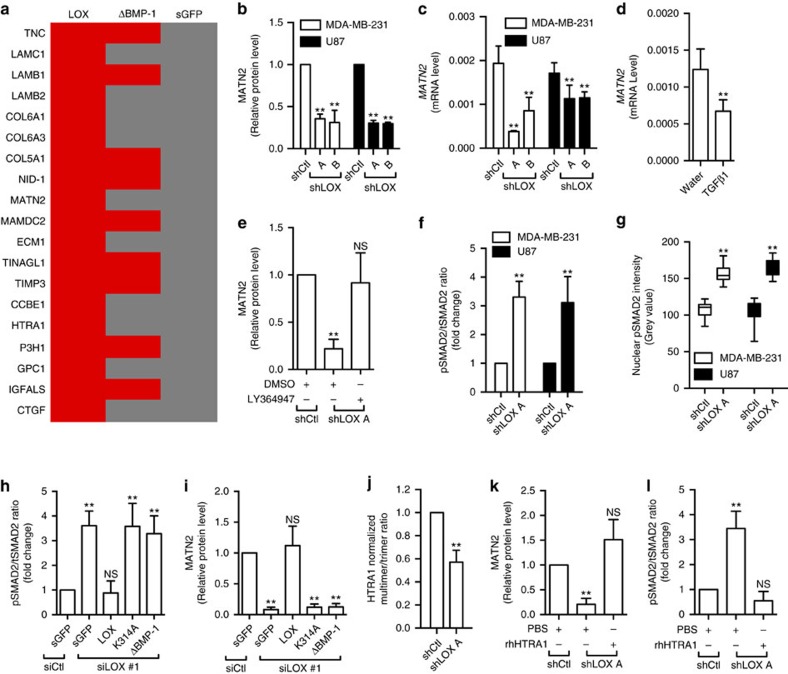
LOX regulates MATN2 by inhibiting TGFβ1 signalling. (**a**) Binding of extracellular proteins to LOX-GFP (LOX), LOX^ΔBMP-1^-GFP (ΔBMP-1) or secreted GFP (sGFP) in MDA-MB-231 culture medium. Red: binding; Grey: no binding. (**b**) MATN2 protein and (**c**) *MATN2* mRNA level in control (shCtl) or LOX-depleted (shLOX A,B) MDA-MB-231 or U87 cells. (**d**) *MATN2* mRNA expression in MDA-MB-231 cells treated with water or TGFβ1. (**e**) MATN2 protein level in control (shCtl) or LOX-depleted (shLOX A) MDA-MB-231 cells treated with DMSO or LY364947. (**f**) SMAD2 activation in control (shCtl) or LOX-depleted (shLOX A) MDA-MB-231 and U87 cells. All data in **b**–**f** are represented as mean±s.d. from three independent experiments. **P<0.01; NS, not significant, Student's *t*-test. (**g**) Nuclear intensity of pSMAD2 in control (shCtl) or LOX-depleted (shLOX A) MDA-MB-231 and U87 cells. Data is represented as min–max from 30 randomly selected cells. **P<0.01, Student's *t*-test. (**h**) SMAD2 activation, and (**i**) MATN2 protein expression in control (siCtl) or LOX-depleted (siLOX #1) MDA-MB-231 and U87 cells expressing secreted GFP (sGFP), LOX-GFP (LOX), LOX^K314A^-GFP (K314A) or LOX^ΔBMP-1^-GFP (ΔBMP-1). (**j**) Quantification of HTRA1 multimer formation as detected by native PAGE in control (shCtl) or LOX-depleted (shLOX A) MDA-MB-231 cells. All data in **h**–**j** are represented as mean±s.d. from four independent experiments. **P<0.01, NS, not significant, Student's *t*-test. (**k**) MATN2 protein level and (**l**) SMAD2 activation in PBS or rhHTRA1 treated control (shCtl) or LOX-depleted (shLOX A) MDA-MB-231 cells. All data in **k**,**l** are represented as mean±s.d. from three independent experiments. **P<0.01, NS, not significant, Student's *t*-test.

To investigate how secreted LOX regulates the EGFR, we sought to identify extracellular proteins that bind to LOX by immunoprecipitating LOX-GFP, LOX^ΔBMP-1^-GFP and sGFP from the culture medium of intact MDA-MB-231 cells. The co-precipitated proteins were analysed by mass-spectrometry, revealing 19 proteins that bind to wild-type LOX-GFP but not to sGFP (Fig. 3a). Notably, 9 of these proteins also bind to LOX^ΔBMP-1^-GFP, leaving 10 (LAMC1, LAMB2, COL6A1, COL6A3, MATN2, ECM1, CCBE1, HTRA1, GPC1, CTGF) that bind specifically to wild-type LOX. Considering that we established that LOX regulates the EGFR, we were particularly intrigued that amongst these is MATN2, which contains 10 EGF-like domains (Supplementary Fig. 3a), so we focused subsequent studies on this protein. We confirmed that MATN2 bound to LOX-GFP and LOX^K314A^-GFP but not LOX^ΔBMP-1^-GFP, confirming that MATN2 bound to mature LOX (Supplementary Fig. 3b).

Intriguingly, we find that LOX depletion by shRNA results in a reduction in MATN2 protein expression in MDA-MB-231 and U87 cells (Fig. 3b; Supplementary Fig. 3c), and confirm this reduced MATN2 accumulation in the extracellular space (Supplementary Fig. 3d,e). Similarly, we find that LOX depletion causes reduced MATN2 accumulation in the cell-derived matrix of BJ dermal fibroblasts (Supplementary Fig. 3f–j). Surprisingly, we find that LOX depletion downregulates MATN2 mRNA (Fig. 3c).

Thus, in addition to binding to MATN2, we demonstrate that LOX also regulates MATN2 mRNA expression, and as it had previously been reported that LOX suppressed TGFβ1 signalling^15^,^16^, and separately that TGFβ1 downregulated MATN2 mRNA^20^, we tested if LOX regulates MATN2 through TGFβ1 signalling. We confirm that addition of TGFβ1 to cells downregulates MATN2 mRNA (Fig. 3d), and that the TGFβ type I receptor inhibitor LY364947 restores MATN2 expression in LOX-depleted MDA-MB-231 cells (Fig. 3e; Supplementary Fig. 3k). We further demonstrate that the TGFβ1 effector SMAD2 is activated when LOX is depleted in MDA-MB-231 and U87 cells (Fig. 3f; Supplementary Fig. 3l) and that phospho-SMAD2 (pSMAD2) accumulates in the nucleus of cells when LOX is depleted (Fig. 3g; Supplementary Fig. 3m), demonstrating that TGFβ1 signalling is activated by LOX depletion. Importantly, the activation of TGFβ1 signalling and accompanying suppression of MATN2 protein expression that occurs when endogenous LOX is depleted are both recovered by expression of wild type LOX-GFP, but not by sGFP, LOX^K314A^-GFP or LOX^ΔBMP-1^-GFP (Fig. 3h,i; Supplementary Fig. 3n). Thus, we conclude that active LOX regulates MATN2 expression through TGFβ1 signalling.

### LOX regulates TGFβ1 signalling through HTRA1

It is reported that LOX regulates TGFβ1 signalling^15^,^16^, but through unknown mechanisms. We do not find evidence of LOX bound to TGFβ1 (Fig. 3a), but note that HTRA1 (high-temperature requirement A serine peptidase 1), a secreted protease that silences TGFβ signalling by degrading TGFβ family proteins^21^,^22^, does bind to LOX-GFP (Fig. 3a). We confirm HTRA1 binding to LOX-GFP by western blotting, but note that it does not bind to LOX^K314A^-GFP or LOX^ΔBMP-1^-GFP (Supplementary Fig. 3o), suggesting that HTRA1 binding to LOX is dependent on LOX maturation and catalytic activity.

HTRA1 is a trimer that is activated by the formation of higher-order multimers^23^, so we used native polyacrylamide gel electrophoresis to examine HTRA1 in the medium from our cells. We observe a high proportion of multimers in the extracellular space of control cells, and these are substantially reduced when LOX was depleted (Fig. 3j; Supplementary Fig. 3p). Critically, when LOX is depleted the addition of recombinant human HTRA1 (rhHTRA1) rescues MATN2 expression (Fig. 3k; Supplementary Fig. 3q) and re-inhibits SMAD2 (Fig. 3l; Supplementary Fig. 3q).

### MATN2 potentiates EGFR activation by EGF

**Figure 4:**
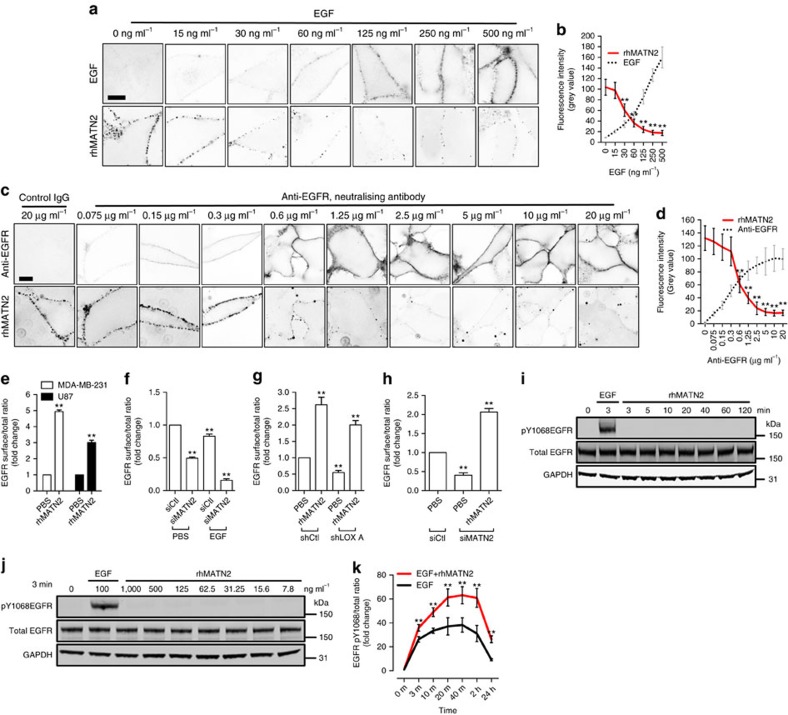
MATN2 traps EGFR at the cell surface. (**a**) Confocal photomicrographs (inverted images) of rhMATN2 (500 ng ml^−1^) binding to PBS or EGF-treated MDA-MB-231 cells at 4 °C at the indicated EGF concentrations. Scale bar, 10 μm. (**b**) Quantification of the fluorescence intensity from images in **a**. (**c**) Confocal photomicrographs (inverted images) of rhMATN2 (500 ng ml^−1^) binding to control IgG or anti-EGFR-treated MDA-MB-231 cells at the indicated anti-EGFR antibody concentrations. Scale bar, 10 μm. (**d**) Quantification of the fluorescence intensity from images in **c**. All data in **b**,**d** are represented as mean±s.d. from 50 images for each data point. **P<0.01, Student's *t*-test. (**e**) Surface EGFR level in PBS or rhMATN2 treated (500 ng ml^−1^) MDA-MB-231 and U87 cells. (**f**) Surface EGFR level in PBS or EGF-treated control (siCtl) and MATN2-depleted (siMATN2) MDA-MB-231 cells. (**g**) Surface EGFR level in PBS or rhMATN2 treated control (shCtl) and LOX-depleted (shLOX A) MDA-MB-231 cells. (**h**) Surface EGFR level in PBS or rhMATN2-treated control (siCtl) and MATN2-depleted (siMATN2) MDA-MB-231 cells. (**i**) Western blots showing pY1068EGFR, total EGFR and GAPDH in EGF stimulated (100 ng ml^−1^, 3 min, positive control) or rhMATN2 stimulated (500 ng ml^−1^, indicated time points) MDA-MB-231 cells. (**j**) Western blots showing pY1068EGFR, total EGFR and GAPDH in MDA-MB-231 cells stimulated with increasing concentrations of rhMATN2 for 3 min. 100 ng ml^−1^ EGF was used as positive control. (**k**) EGFR activation in MDA-MB-231 cells treated with EGF, or EGF and rhMATN2 at indicated time points. All data in **e**–**h** and **k** are represented as mean±s.d. from three independent experiments. **P<0.01, Student's *t*-test.

Our data show that LOX regulates MATN2 expression through HTRA1 and TGFβ signalling, so we next examined how MATN2 regulates EGFR signalling. As noted, MATN2 protein has ten EGF-like domains, which occupy most of the protein (Supplementary Fig. 3a), so we tested if it could bind to cell surface EGFR. We find that recombinant human MATN2 (rhMATN2) binds to the surface of cells (Supplementary Fig. 4a), but importantly, this cell surface binding is competed away by EGF (Fig. 4a,b). Moreover, an EGFR neutralizing antibody also competes for rhMATN2 binding to the cell surface (Fig. 4c,d). Notably, addition of rhMATN2 increases EGFR levels at the surface of MDA-MB-231 and U87 cells (Fig. 4e; Supplementary Fig. 4b) and depletion of endogenous MATN2 (Supplementary Fig. 4c) causes a loss of EGFR from the cell surface under basal and EGF stimulated conditions (Fig. 4f; Supplementary Fig. 4d). Critically, the addition of rhMATN2 to the cell culture medium rescues EGFR retention at the cell surface when LOX is depleted (Fig. 4g; Supplementary Fig. 4e), and it rescues cell surface EGFR when MATN2 is depleted (Fig. 4h; Supplementary Fig. 4f). Note that rhMATN2 does not activate EGFR (Fig. 4i,j), but it strongly enhances EGFR activation when the cells are stimulated by EGF (Fig. 4k, Supplementary Fig. 4g). Thus, we conclude that MATN2 binds to, but does not activate the EGFR; rather MATN2 potentiates EGFR activation stimulated by EGF.

### MATN2 potentiates EGF-driven cell proliferation

**Figure 5:**
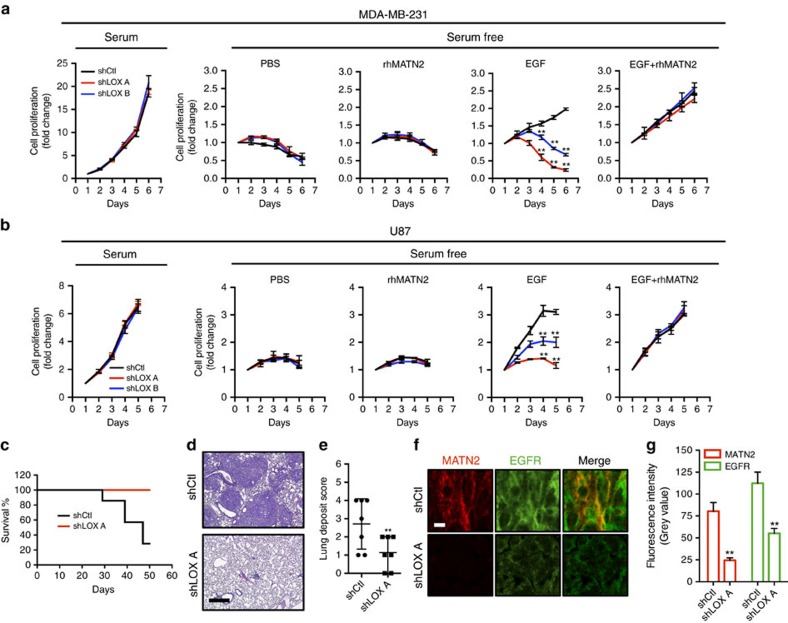
LOX controls EGF-dependent cell proliferation *in vitro* and tumour growth *in vivo*. (**a**,**b**) Graphs showing cell proliferation in control (shCtl) and LOX-depleted (shLOX A,B) MDA-MB-231 (**a**) and U87 (**b**) cells grown in 10% FBS (serum) or serum-free medium (serum free) and treated with PBS, rhMATN2 (500 ng ml^−1^) and EGF (50 ng ml^−1^) as indicated. All data are represented as mean±s.d. from four independent experiments. **P<0.01, Student's *t*-test. (**c**) Kaplan–Meier survival plot for mice following tail vein injection of control (shCtl) or LOX-depleted (shLOX A) MDA-MB-231 cells. Seven mice were used in each group. (**d**) H&E staining of lungs from mice in **c**. Scale bar, 500 μm. (**e**) Quantification of lung deposits in mice. Data is represented as mean±s.d. from seven samples. **P<0.05, Mann–Whitney analysis. (**f**) Photomicrographs for MATN2 (red) and EGFR (green) in lung deposits. Scale bar, 10 μm. (**g**) Quantification of samples in **f**. Data is represented as mean±s.e.m. from six samples. **P<0.01, Student's *t*-test.

Next, we examine how this pathway regulates cell proliferation. First, we show that in 10% serum, the proliferation of MDA-MB-231 and U87 (serum) is not affected by LOX depletion (Fig. 5a,b). Next, we confirm that these cells do not grow in serum free medium whether or not LOX is depleted (Fig. 5a,b). We also show that rhMATN2 does not rescue cell growth in serum free medium whether or not LOX is depleted (Fig. 5a,b). Importantly, EGF can support cell growth in serum free medium, but when LOX is depleted, EGF can no longer rescue cell growth (Fig. 5a,b). Thus, in serum-free medium, EGF requires LOX to support cell growth, but critically, when LOX is depleted, the addition of rhMATN2 to the medium can rescue cell growth driven by EGF (Fig. 5a,b). Thus, consistent with our biochemical observations, when proliferation is inhibited because LOX is depleted, EGF plus rhMATN2 rescue cell growth.

We then examined the effects of this signalling pathway *in vivo* by injecting MDA-MB-231 cells into the tail vein of immunocompromised mice. Within 50 days, 5/7 animals that received shRNA control cells were killed in accordance with licence guidelines^24^, whereas all animals that received LOX-depleted cells remained healthy (Fig. 5c). The mice that received shRNA control cells had substantial tumour burden in the lungs, whereas lung tumour burden was substantially lower in mice that received LOX-depleted cells (Fig. 5d,e; Supplementary Table 1). Critically, we observe significantly less staining of MATN2 and EGFR in the plasma membranes of the cells in the LOX-depleted tumours than in the cells of shRNA control tumours (Fig. 5f,g).

### Discovery of a potent oral LOX inhibitor CCT365623

**Figure 6:**
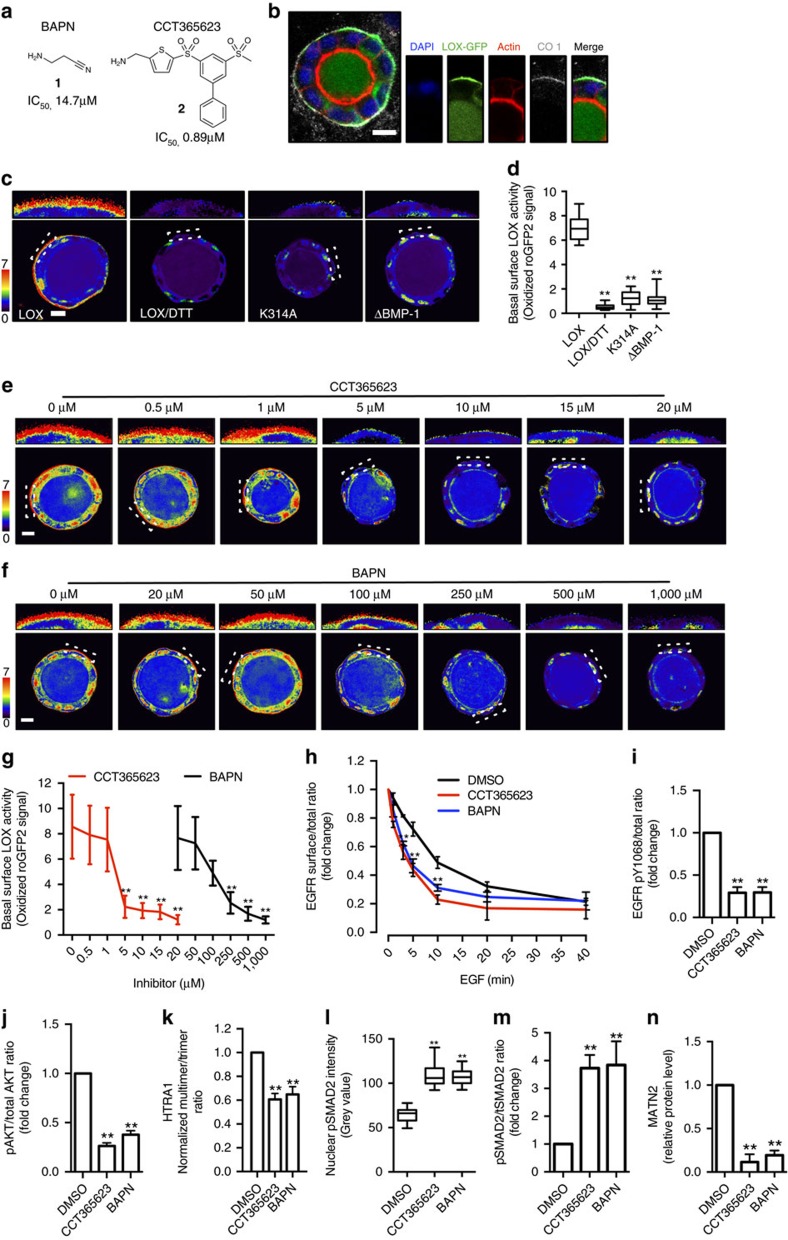
CCT365623 and BAPN inhibits LOX enzyme activity reported by a LOX biosensor. (**a**) Chemical structures and biochemical IC_50_ for CCT365623 and BAPN. (**b**) Confocal photomicrographs of LOX-GFP (green) in a MDCK cyst co-stained for DAPI (blue), actin (red) and collagen type 1 (CO1) (grey). Scale bar, 10 μm. (**c**) Confocal ratio images showing enzyme activities of indicated LOX-roGFP2 biosensors. Negative control: DTT-treated LOX-roGFP2 cysts. Scale bar, 10 μm. (**d**) Quantification of LOX activities in **c**. Data are represented as min–max from 20 MDCK cysts. **P<0.01, Student's *t*-test. (**e**,**f**) Confocal ratio images showing LOX activity in LOX-roGFP2 MDCK cysts treated with increasing concentrations of CCT365623 or BAPN. Scale bars, 10 μm. (**g**) Dose–response curves of CCT365623 and BAPN using LOX-roGFP2 biosensor from **e**,**f**. Data are represented as min–max from 20 MDCK cysts per concentration. **P<0.01, Student's *t*-test. (**h**) Surface EGFR retention, (**i**) EGFR activation and (**j**) AKT activation in DMSO, CCT365623 or BAPN treated MDA-MB-231 cells. All data in **h**–**j** are represented as mean±s.d. from three independent experiments. **P<0.01, Student's *t*-test. (**k**) Quantification of extracellular HTRA1 Multimer/Trimer ratio in DMSO, CCT365623 or BAPN-treated MDA-MB-231 cells. Data are represented as mean±s.d. from three independent experiments. **P<0.01, Student's *t*-test. (**l**) Quantification of nuclear pSMAD2 in DMSO, CCT365623 or BAPN-treated MDA-MB-231 cells. Data are represented as min–max from 30 cells per condition. **P<0.01, Student's *t*-test. (**m**) SMAD2 activation, (**n**) MATN2 protein level in DMSO, CCT365623 or BAPN-treated MDA-MB-231 cells. All data in **m**,**n** are represented as mean±s.d. from three independent experiments. **P<0.01, Student's *t*-test.

Next, we used LOX pharmacological inhibitors to test the therapeutic potential of our findings. Most studies in the field rely on a single LOX inhibitor, BAPN (β-aminopropionitrile) (compound **1** in Fig. 6a). We developed complementary compound, CCT365623 (compound **2** in Fig. 6a), a LOX inhibitor from a distinct structural class that is ∼16-fold more potent than BAPN (Fig. 6a). To confirm that CCT365623 inhibits LOX in living cells, we created a biosensor based on the observation that LOX generates H_2_O_2_ as a by-product of its catalytic activity and surmized that it should be possible to detect this molecule using a redox-sensitive version of GFP (roGFP2) (Supplementary Fig. 5a)^25^,^26^.

To create our biosensor, we fuse roGFP2 to the C terminus of LOX (LOX-roGFP2). We express the biosensor in MDCK cells because when these cells are grown as cysts in matrigel, they secrete a layer of the LOX substrate collagen type 1 (CO1) on their basal surface. First, we confirm that standard LOX-GFP co-localizes with this basal layer of collagen type 1 in MDCK cysts (Fig. 6b). Next, we show that LOX-roGFP2 generates a robust signal on the basal surface of the cysts that is quenched when the cysts are treated with the reducing agent dithiothreitol (DTT) (Fig. 6c,d). Moreover, we do not observe a signal with biosensors made with catalytically inactive LOX (LOX^K314A^-roGFP2) or the version of LOX resistant to BMP-1-mediated cleavage (LOX^ΔBMP-1^-roGFP2) (Fig. 6c,d). Thus, our biosensor is able to measure LOX activity in MDCK cysts, and we show that in this living cell system CCT365623 inhibits LOX at ∼5 μM, whereas BAPN inhibits LOX at ∼250 μM (Fig. 6e–g).

Next, we show that CCT365623 and BAPN both reduce EGFR retention at the cell surface (Fig. 6h; Supplementary Fig. 5b,c) and that they both suppress EGFR and AKT phosphorylation driven by EGF (Fig. 6i,j; Supplementary Fig. 5d). We show also that both compounds inhibit HTRA1 multimer formation, that they activate SMAD2 and downregulate MATN2 (Fig. 6k–n; Supplementary Fig. 5e–h). Note that CCT365623 is effective at 50-fold lower concentrations than BAPN (5 versus 250 μM), but that the results from both inhibitors are consistent with our genetic experiments.

### CCT365623 inhibits tumour growth

Next, we tested CCT365623 *in vivo*. First, we show that CCT365623 displays good stability in mouse liver microsomes and does not inhibit the cardiac potassium channel hERG. It displays excellent selectivity (over 350-fold) for LOX over amine oxidase A and B (MAO-A, MAO-B) and is over 1,000-fold more selective for LOX over serum diamine oxidase (DAO) (Supplementary Table 2), another copper and quinone-cofactor containing amine oxidase that prevents scombroid poisoning by scavenging extracellular histamine. BAPN also displays generally favourable properties, but in addition to being significantly less potent than CCT365623 it is only ∼14-fold more selective for LOX over DAO (Supplementary Table 2). Critically, CCT365623 is orally available, is extremely well tolerated (Supplementary Fig. 6a), and has good pharmacokinetic properties (Supplementary Table 3).

**Figure 7:**
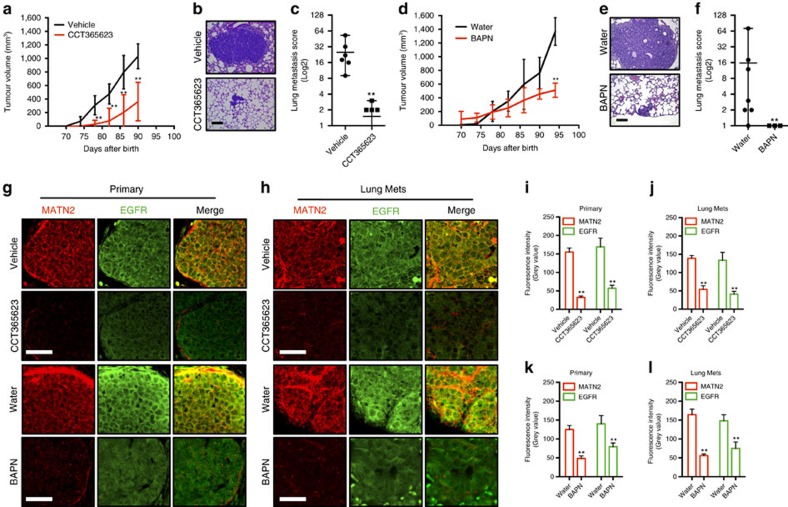
Chemical inhibition of LOX blocks tumour growth and metastasis. (**a**) Tumour growth in vehicle or CCT365623-treated MMTV-PyMT mice. Data are represented as mean±s.d. from six mice per group. **P<0.01, Student's *t*-test. (**b**) H&E stained lungs from vehicle or CCT365623 treated MMTV-PyMT mice. Scale bar, 100 μm. (**c**) Quantification of lung metastasis in mice from **a**. All data are presented as mean±range, *n*=6 animals. **P<0.05, Mann–Whitney analysis. (**d**) Tumour growth in water or BAPN-treated MMTV-PyMT mice. Data are represented as mean±s.d. from seven mice per group. **P<0.01, Student's *t*-test. (**e**) H&E stained lungs from water or BAPN-treated MMTV-PyMT mice. Scale bar, 100 μm. (**f**) Quantification of lung metastasis in mice from **d**. All data are presented as mean±range, *n*=7 animals. **P<0.05, Mann–Whitney analysis. (**g**,**h**) Confocal photomicrographs of MATN2 (red) and EGFR (green) staining in primary and metastatic tumours from vehicle, CCT365623, water or BAPN-treated MMTV-PyMT mice. Scale bars, 20 μm. (**i**–**l**) Quantification of MATN2 and EGFR staining in primary or metastatic tumours from **g** and **h**. All data are represented as mean±s.e.m. Samples from six vehicle or CCT365623 and seven water or BAPN-treated mice were analysed. **P<0.01, Student's *t*-test.

We tested CCT365623 in a mouse model of spontaneous breast cancer that metastasizes to the lungs^27^. In this model the primary breast tumours become palpable when the mice are 70–75 days old, so we administered CCT365623 by oral gavage (70 mg kg^−1^ per day) starting when the mice were 70 days old. CCT365623 significantly delays the development of the primary tumours and also suppresses metastatic lung burden in the animals (Fig. 7a–c; Supplementary Table 4). When BAPN is administered using this protocol, it does not inhibit tumour development (Supplementary Fig. 6b), but when it is administered *ad libitum* in the drinking water (1% w/v), we observe a significant delay in primary tumour development and a reduction in metastatic lung tumour burden (Fig. 7d–f; Supplementary Table 4). Notably, CCT365623 and BAPN elicit a significant reduction in MATN2 protein levels in both the primary and metastatic lung tumours and this is accompanied by the loss of EGFR from the plasma membranes of the cells in both the primary and metastatic tumours (Fig. 7g–l).

## Discussion

**Figure 8:**
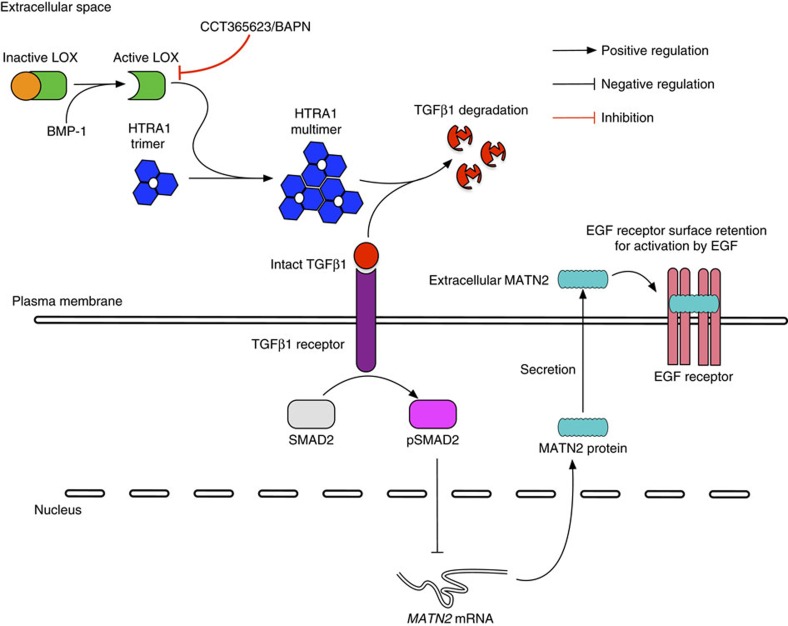
Model describing LOX regulation of EGFR signalling. The diagram summarizes the regulation of EGFR signalling by LOX through HTRA1 and MATN2 in cells.

Previously, LOX was shown to regulate integrin and consequently SRC signalling by modulating ECM stiffness^8^,^10^. We confirm that LOX regulates SRC but importantly, we also describe a previously unidentified function of LOX in regulating the EGFR. On the basis of our data, we propose a model whereby LOX activates the secreted protease HTRA1, which inhibits TGFβ1 signalling, causing increased expression of the EGF-like domain containing protein MATN2, which then enhances EGFR signalling to drive tumour growth and metastasis (Fig. 8).

To date, the most commonly used pharmacological inhibitor of LOX has been BAPN, an organic compound that is active in the mid-micromolar range, but which does not have drug-like properties and is thus not suitable for further pharmacological development. Here, we describe CCT365623, a LOX inhibitor that has clinical development potential. We confirm that CCT365623 inhibits purified LOX *in vitro*. We also developed a biosensor that measures LOX activity against its natural substrates and show that CCT365623 inhibits this biosensor in living cells grown in a 3D culture system. Moreover, like BAPN or genetic ablation of LOX in cells, CCT365623 disrupts HTRA1 multimerization, activates TGFβ1 signalling, suppresses MATN2 expression, suppresses EGFR surface retention, and suppresses EGFR signalling. Finally, like BAPN or genetic ablation of LOX, CCT365623 downregulates MATN2 and inhibits EGFR plasma membrane localization in tumours, and it suppresses tumour growth and metastasis in mice.

Thus, we show that CCT365623 is a LOX inhibitor both biochemically against the isolated enzyme and in living cells. *In vitro* and *in vivo* it recapitulates the biological effects of both BAPN and LOX depletion. The catalytic domains of LOX and the other family proteins (LOX-like protein1–4) are highly conserved, and some of these are known to be inhibited by BAPN^28^. It is therefore likely that CCT365623 inhibits these LOX-like enzymes and an assessment of the selectivity against other unrelated enzymes is ongoing. However, in the absence of crystal structures, we do not understand how BAPN or CCT365623 bind to LOX, and so it is not possible to generate LOX mutants that are resistant to these agents for *in vitro* and *in vivo* testing. Nevertheless, our data suggest that like BAPN, CCT365623 is a LOX inhibitor that modulates the biological functions of LOX in cells and has anti-tumour activity in mice. Moreover, CCT365623 is more potent than BAPN and it displays superior selectivity against serum DAO. It has promising drug-like properties with good liver microsome stability, oral bio-availability, robust pharmacokinetic properties and it is well tolerated in mice. Therefore, CCT365623 is a useful inhibitor to help improve our understanding of LOX biology.

HTRA1 is a trimer that is known to form higher-order complexes (multimers) that drive its activation and critically, we show that LOX induces HTRA1 multimerization. Using GFP-trapping followed by mass spectrometry and western blotting, we show that LOX binds to HTRA1. In cells we show that LOX depletion by RNA interference, or its inhibition by CCT365623 or BAPN impairs the formation of the HTRA1 multimers. This suggests that LOX activity is needed to induce HTRA1 multimerization, and accordingly we show that HTRA1 binds to wild-type LOX but not catalytically inactive LOX, or LOX that cannot be cleaved to the mature active form by BMP-1.

We show that when LOX is depleted, TGFβ1 signalling is activated and MATN2 expression is reduced. Similarly, when LOX is inhibited by CCT365623 or BAPN, TGFβ1 signalling is activated and MATN2 expression reduced. Moreover, treatment of the cells with TGFβ1 suppresses MATN2 expression, whereas inhibition of the TGFβ1R increases MATN2 expression. Critically, addition of recombinant HTRA1 is sufficient to suppress TGFβ1 signalling and induce MATN2 expression when LOX is depleted. Altogether these data suggest that LOX activates HTRA1 by inducing higher-order complexes and that HTRA1 inhibits TGFβ1 signalling to increase MATN2 expression.

We show that MATN2 increases EGFR activation by EGF. Although we were unable to show direct binding of MATN2 to EGFR *in vitro*, we show that EGF and an EGFR neutralizing antibody compete with MATN2 for binding to the cell surface, suggesting direct binding of MATN2 to the EGFR. In support of this is the observation that MATN2 contains 10 EGF-like domains, and accordingly MATN2 depletion reduces EGFR retention at the cell surface, whereas addition of recombinant MATN2 to cells increases EGFR retention at the cell surface. Critically, when LOX is depleted EGFR retention at the cell surface is reduced, but this was reversed by addition of recombinant MATN2 to the culture medium. Surprisingly, despite this evidence for MATN2 binding to EGFR, MATN2 does not activate the receptor, but rather it enhances its activation by EGF. These data suggest that the function of MATN2 is to trap EGFR at the cell surface, thereby increasing receptor numbers and present the receptors to EGF for signalling. Notably, *MATN2* is amplified or over expressed in 20% breast cancers and there is a significant tendency for this to co-occur with *EGFR* gene alterations in invasive breast carcinomas^29^,^30^. Clearly, it will be interesting to determine if MATN2 regulates other EGFR family members and if it regulates heterodimers of EGFR and other family members.

To conclude, we have identified a molecular function of extracellular LOX in regulating the EGFR. We identify MATN2 as the key mediator of this pathway and demonstrate that LOX regulates MATN2 expression through HTRA1 and TGFβ1 signalling. We have discovered CCT365623, a LOX inhibitor with great therapeutic promise that is also valuable for studying LOX mechanism *in vitro* and *in vivo*. We show that pharmaceutical inhibition of LOX disrupts this signalling pathway, reducing EGFR signalling and delaying tumour progression. CCT365623 and its analogues are undergoing clinical development for use in LOX driven cancers.

## Methods

### General procedures

All chemicals were purchased from Sigma unless otherwise stated.

### Cell culture and transfection

Cell lines used in this study were purchased from American Type Culture Collection (ATCC), routinely monitored by PCR to ensure they were mycoplasma free and authenticated by STR profiling. Cell culture reagents were purchased from Life Technologies. MDA-MB-231, U87, BJ and MDCK cell lines were cultured in Dulbecco's Modified Eagle Medium (DMEM) supplemented with 10% fetal bovine serum (FBS) and 1% penicillin/streptomycin solution (pen/strep). For stimulation studies, serum starved cells were treated with 100 ng ml^−1^ EGF (Peprotech) or 1 μg ml^−1^ rhMATN2 (R&D). For TGFβ1 treatment, TGFβ1 (R&D) was dissolved in water and added to culture medium at a final concentration of 5 ng ml^−1^ for overnight treatment of MDA-MB-231 cells. MDA-MB-231, U87 and MDCK cells were transfected using lipofectamine 3000 according to manufacturer's instructions. BJ fibroblasts were infected with lentiviruses (Sigma). Cells were selected with G418 (5 mg ml^−1^, Life Technologies) or puromycin (3 μg ml^−1^, Invivogen).

To generate MDCK cysts, cells were cultured on solidified Matrigel (Corning) for 10 days in DMEM with 10% FBS supplemented with 2% Matrigel. MDA-MB-231 and U87 cells were cultured on thick collagen gels formed from acid extracted rat tail collagen type 1 (Corning) to observe matrix deposition, or embedded in collagen gels for protein analysis. BJ fibroblasts were plated on gelatin (Sigma) coated glass bottomed dishes (MatTek) for 7 days to allow formation of cell-derived matrices.

### RNA interference

To generate stable LOX-depleted MDA-MB-231 and U87 cell lines, shLOX A (TTGTTATTGAAAACAGTCC, V3LHS_406838) targeting human LOX, and shLOX B (ACATCTGTAATATCAATCC, V3LHS_348880) targeting mouse and human LOX was used and GIPZ non-silencing shRNA (all from Dharmacon) was used as a control. Stable LOX depletion in BJ fibroblasts was achieved using a lentivirus (shLOX C) containing human-specific LOX targeting shRNA (CGACAACCCTTATTACAACTA, TRCN0000045991) and pLKO.1 non-targeting shRNA was used as control (both from Sigma).

For transient LOX depletion, MDA-MB-231 or U87 cells were transfected with human specific siLOX#1 (AAGCTGGCTACTCGACATC, SI00036120), or mouse and human specific siLOX#2 (CTGCACAATTTCACCGTAT, SI00036113) and for transient MATN2 depletion, siMATN2 (ATGCCGAAGACTTCAGCACAA, SI04169795) was used. AllStars negative control siRNA (all from Qiagen) served as control.

### Western blotting

Proteins were extracted from cultured cells using cell lysis buffer (Cell Signalling Technology) with the addition of protease and phosphatase inhibitor cocktails (Pierce). Protein concentrations were measured using Pierce 660 nm protein assay reagent and a SpectaMax M5 plate reader. Equal amounts of proteins were separated using NuPAGE 4–12% Bis-Tris gels with NuPAGE MOPS SDS running buffer (Life Technologies). Separated proteins were transferred to nitrocellulose membranes using a Trans-Blot turbo transfer system (Bio-Rad). Following blocking with Odyssey blocking buffer (Li-COR), membranes were incubated with two primary antibodies of different species at 4 °C over night. The following primary antibodies were used, anti-human LOX (1:500; Sigma, L4794) and anti-tubulin (1:5,000; Sigma, T9026), anti-human MATN2 (1:500; R&D, AF3044), anti-EGFR (1:2,000; Cell Signalling Technology, 4267) and anti-pY1068EGFR (1:1,000; Cell Signalling Technology, 3777), anti-GFP (1:2,000; Cell Signalling Technology, 2956) and anti-GAPDH (1:10,000; Millipore, CB1001-500UG). Corresponding fluorescence secondary antibodies (1:5,000; Li-COR) were used to visualize the protein of interest on an Odyssey CLx infrared imaging system (Li-COR). Quantification of band fluorescent intensity was achieved using Li-COR ImageStudio. All protein band fluorescent intensity quantifications were normalized to its internal controls (GAPDH or Tubulin). To determine phospho/total protein ratio, band fluorescent intensity was first normalized to corresponding internal controls. Then the ratio was calculated. To determine surface EGFR/total EGFR ratio, a cell lysis sample was divided, so that equal amount of proteins was used for surface EGFR isolation and for total EGFR detection. The surface EGFR/total EGFR ratio was then calculated: surface EGFR/total EGFR/GAPDH.

Homotrimeric HTRA1 in cell culture medium was resolved using Blue NativePAGE (Life Technologies). Extracellular proteins in 15 ml serum-free cell culture medium with protease inhibitors added was first concentrated using Vivaspin protein concentrator spin columns (GE Healthcare Life Sciences). Proteins in concentrated culture medium were then separated using NativePAGE 3–12% Bis-Tris Gel and NativePAGE running buffer (Life Technologies). Separated proteins were transferred to nitrocellulose membranes using a Trans-Blot turbo transfer system (Bio-Rad) as normal. This is then followed by standard blotting procedures as described above. Anti-human HTRA1 antibody (1:500; R&D, MAB2916) was then used to detect native HTRA1 proteins.

Uncropped full scans of blots are presented in Supplementary Fig. 7.

### Quantitative real time PCR

RNA was extracted with Trizol (Life Technologies) according to the manufacturer's instructions. RNA was reverse-transcribed to generate complementary DNA using M-MLV Reverse Transcriptase (Sigma). Real-time qRT-PCR was performed with FastStart Universal Probe Master *(Rox)* (Roche) and TaqMan Gene Expression Assay probes on an Applied Biosystems 7900HT Fast Real Time machine. Relative *MATN2* (hs00242753_m1, Life Technologies) expression was calculated using the Δ*C*t method and housekeeping genes (*GAPDH*) as an internal control.

### Immunofluorescence

All samples were fixed in 4% paraformaldehyde (EMS). Fixed cells were subjected to incubation with primary antibodies followed by fluorescence secondary antibodies (Life Technologies) to visualize extracellular proteins. For visualization of intracellular proteins, fixed cells were permeabilized using 0.1% Triton X-100 (Sigma) before primary and secondary antibody incubations. The following primary antibodies were used at 1:100 dilutions, anti-collagen type1 (Abcam, ab34710), anti-human MATN2 (R&D, AF3044) and anti-pSMAD2 (Millipore, AB3849). Alexa Fluor 594 Phalloidin (Life Technologies) was used for actin staining. DAPI or Hoechst (Sigma) was used to stain DNA.

### Confocal imaging and imaging analysis

All photomicrographs were taken with a Leica TCS SP8 X confocal system. Where necessary, fluorescence intensity was measured as grey values using Fiji. Statistical significance of grey value differences between samples was tested using Student's *t*-test. For LOX biosensor imaging, the oxidized biosensor was excited using a 405 nm laser, while the reduced biosensor was excited with a 488 nm laser. Emission of the biosensor was recorded at 500–530 nm using sequential scans. Ratio images were generated following a published protocol^31^. Note, while the published protocol generates YFP/CFP ratio images, we used it to generate oxidized/reduced (oxidized roGFP2 signal) ratio images.

To image extracellular MATN2, MDA-MB-231 or U87 cells were culture on collagen gels as described in cell culture and transfection section. BJ fibroblasts CDM was also generated as described in cell culture and transfection section. Samples were then fixed and stained with anti-human MATN2 and Hoechst. For each sample, 15 random fields of views were taken from three repeats. MATN2 fluorescence intensity per image was calculated using Fiji. The number of cells present in each image were counted based on DAPI staining and GFP expression, so that the MATN2 fluorescence intensity could be normalized to cell number.

To quantify nuclear pSMAD2 fluorescence intensity, cell nucleus was first selected based on DAPI staining. pSMAD2 fluorescence intensity in the selected region was then measured using Fiji. 30 cells were randomly selected per condition.

In order to quantify the fluorescence intensity in tumour samples, 3–5 images were acquired per tumour section depending on the actual size. An average fluorescence intensity of MATN2 and EGFR staining were then calculated using values from individual images. This average florescence intensity was used to represent MATN2 and EGFR levels in a given tumour.

### PathScan RTK signalling antibody array

Cell lysates of MDA-MB-231 cells cultured in 3D collagen gel were collected using cell lysis buffer (Cell Signalling Technology) with protease and phosphatase inhibitor cocktails (Pierce). PathScan RTK signalling antibody arrays (Cell Signalling Technology, 7949) were used according to manufacturer's instructions using equal amounts of proteins. The fluorescent readout was recorded on an Odyssey CLx infrared imaging system (Li-COR). Quantification of the array fluorescent intensity was achieved using Li-COR ImageStudio.

### AKT activation assay

Serum starved MDA-MB-231 or U87 cells were stimulated with 100 ng ml^−1^ EGF. Cells were then lysed as usual. Equal amounts of proteins were used for AKT activation analysis using a pS473AKT/total AKT ELISA kit (Abcam) according to manufacturer's instructions.

### Labelling and detection of surface EGFR

Following 100 ng ml^−1^ EGF stimulation, cells were washed with ice-cold PBS and then incubated with ice-cold 0.24 mg ml^−1^ Sulfo-NHS-SS-Biotin (Pierce) in PBS for 30 min at 4 °C. Excess Sulfo-NHS-SS-Biotin was blocked using 50 mM NH_4_Cl in PBS and washed using ice-cold PBS. Cells were lysed and protein concentrations determined as described above. Biotinylated proteins from equal amounts of cell extract were captured at room temperature using NeutrAvidin Agarose beads (Pierce) for 1 h and analysed by western blotting.

### Cloning of LOX expression constructs

Mouse LOX cDNA was purchased from OriGene. Full length LOX cDNA was PCR cloned into pEGFP-N1 (Clonetech), or proGFP2-N1 (Jim Remington, University of Oregon) using the following primers, GAGAGAGCTAGCATGCGTTTCGCCTGGG (forward primer) and TCTCTCCTCGAGATACGGTGAAATTGTGCAGCC (reverse primer). For the insertion into pEGFP-N1 or proGFP2-N1, NheI and XhoI restriction sites were added to forward and reverse primers accordingly. Mutant LOX constructs were made using QuickChange II site-directed mutagenesis kit (Agilent Technologies) following manufacturer's instruction using LOX-GFP as template. To create LOX^K314A^-GFP, the following primer pair was used, GAGTGGCTGAAGGCCACGCAGCAAGCTTCTGTCTGG (sense), and CCAGACAGAAGCTTGCTGCGTGGCCTTCAGCCACTC (antisense). For LOX^ΔBMP-1^-GFP, CAGCCACATAGATCGCCCCTACAAGTACTCCG (sense) and CGGAGTACTTGTAGGGGCGATCTATGTGGCTG (antisense) was used. To generate, roGFP2 versions of LOX mutant constructs, LOX mutant cDNA was transferred from pEGFP-N1 to proGFP2-N1 using NheI and XhoI. Finally, LOX signal peptide was PCR cloned into pEGFP-N1 vector to make a secreted GFP (sGFP) expression construct using GAGAGAGCTAGCATGCGTTTCGCCTGGG (forward primer) and TCTCTCCTCGAGCGGGGCGCAGCGGAGAA (reverse primer).

### Co-immunoprecipitation of extracellular LOX-binding proteins

MDA-MB-231 cells with stable expression of LOX-GFP, LOX^K314A^-GFP, LOX^ΔBMP-1^-GFP and sGFP were used to immunoprecipitate LOX proteins from culture medium. Co-immunoprecipitated proteins were subjected to analysis by mass spectrometry or by western blotting. The band intensity of MATN2 or HTRA1 was normalized to corresponding band intensities of precipitated LOX-GFP proteins as an indication of relative binding strength.

### Identification of extracellular proteins that bind to LOX-GFP

MDA-MB-231 cells with stable expression of LOX-GFP, LOX^K314A^-GFP, LOX^ΔBMP-1^-GFP and sGFP were used to immunoprecipitate LOX proteins from culture medium. For each immunoprecipitation, 45 ml culture medium was collected from three confluent 150 mm cell culture dishes. Protease inhibitor cocktails (Roche) were added immediately following medium collection. Debris in medium was removed by centrifugation. Cleared cell culture medium was then incubated with 50 μl GFP-Trap agarose beads (ChromoTek) overnight at 4 °C. Agarose beads were then washed according to manufacturer's protocol. Immunoprecipitated proteins were separated on NuPAGE 4–12% Bis-Tris gels (Life Technologies) for western blots or staining with SimplyBlue SafeStain (Life Technologies).

For analysis by mass spectrometry, SimplyBlue-stained gel slices were destained with 3 × 20 min changes of 1 ml 200 mM ammonium bicarbonate and 40% (v/v) acetonitrile. Gel slices were dehydrated by the addition of 500 μl acetonitrile for 15 min followed by rehydration in 500 μl of water for a further 15 min. This dehydration–rehydration procedure was performed three times followed by a final dehydration in acetonitrile.

Gel slices were rehydrated in 25 μl of 50 mM ammonium bicarbonate, 9% (v/v) acetonitrile, and 20 ng μl^−1^ sequencing grade trypsin (Sigma) for 20 min. The slices were then covered in 100 μl of 50 mM ammonium bicarbonate and 9% (v/v) acetonitrile and incubated at 37° for 18 h. Following digestion, samples were acidified by the addition of 10 μl of 10% (v/v) formic acid. The isolated supernatant was dried in a vacuum centrifuge at 40 °C for 30 min and the released peptides were resuspended in 2 μl of water and 0.1% trifluoroacetic acid.

LC–MS/MS analyses were performed on an LTQ Orbitrap XL mass spectrometer with EasySpray source coupled to an Ultimate 3000 RSLCnano system (Thermo Scientific). Samples were loaded directly onto the analytical column, PepMap RSLC C18, 2 μm × 75 μm id × 50 cm (Thermo Scientific) maintained at 60 °C. The composition (v/v) of LC buffers was: buffer A—water plus 0.1% formic acid; buffer B—80% acetonitrile, 19.9% water and 0.1% formic acid. Peptides were loaded directly onto the column at a flow rate of 300 nl min^−1^ with an initial mobile phase composition of 1% B. The organic strength was increased linearly from 1 to 22.5% B over 20 min again at 300 nl min^−1^, followed by an increase to 40% B over the next 3 min. A further increase to 80% B over the next 5 min was followed by a hold phase for 5 min before returning to 1% B for 22 min. The mass spectrometer was instructed to perform data dependent acquisition on the top six precursor ions, which were measured in the Orbitrap FTMS detector over the mass range 370–1,200 *m*/*z*, at a nominal resolution of 60,000. MS/MS spectra were acquired in the ion trap under CID conditions with normalized collision energy of 35, isolation width of 3 Th, *Q* value of 0.25 and 30 ms activation time.

### MATN2 cell surface-binding assay

To detect cell surface binding of MATN2, 500 ng ml^−1^ rhMATN2 was incubated with MDA-MB-231 cells in glass bottom dishes (MatTek) for 30 min. Culture medium was then removed. Cells were then washed with ice cold PBS before fixation and staining for immunofluorescence and confocal imaging.

### MATN2 cell surface-binding competition assays

To demonstrate EGFR dependent cell surface binding of MATN2, MDA-MB-231 cells were cultured in glass bottom dishes (MatTek) at 37 °C and serum starved cells were pre-treated with 20 μg ml^−1^ control mouse IgG1 (Millipore, MABC002) or 0.075–20 μg ml^−1^ mouse anti-EGFR, clone LA1 neutralizing antibody (Millipore, 05-101) for 30 min. Overall, 500 ng ml^−1^ rhMATN2 was then added (without diluting the competing antibody) to cells for a further 30 min. Samples were washed in ice cold PBS and fixed for immunofluorescence and confocal imaging. Images were acquired randomly. Experiments were repeated three times, and a total of 50 fields of views were taken per condition. The average fluorescence intensity per condition was then calculated using the 50 images.

EGF was also used to compete with rhMATN2 (R&D) for cell surface binding to EGFR. MDA-MB-231 cells were cultured and serum starved as described above. Before the competition assay, cells were washed with ice cold PBS and left on ice. Either 500 ng ml^−1^ rhMATN2 in PBS or a mixture of 500 μg ml^−1^ rhMATN2 and 15–500 ng ml^−1^ EGF (Alexa Flour 647 EGF, Life Technologies) in PBS was used to treat cells on ice for 30 min. Samples were then washed with ice cold PBS and fixed for immunofluorescence and confocal imaging. Images were acquired randomly. Experiments were repeated three times, and a total of 50 fields of views were taken per condition. The average fluorescence intensity per condition was then calculated using the 50 images.

### Cell proliferation assay

2000 MDA-MB-231 or U87 cells were seeded into 96-well plates and cultured under indicated conditions. Cell proliferation was determined by CellTiter-Glo luminescence (Promage) according to manufacturer's instructions on indicated days. Data were normalized to day 1. Student's *t*-test was used to calculate the statistical significance.

### Animal procedures

All procedures involving animals were performed under licence PPL-70/7701, 70/6730 and 70/7635, in accordance with ARRIVE guidelines and National Home Office regulations under the Animals (Scientific Procedures) Act 1986. Procedures were approved by the Animal Welfare and Ethical Review Bodies (AWERB) of the CRUK Manchester Institute and the Institute of Cancer Research, and tumour volumes did not exceed the guidelines set by the Committee of the National Cancer Research Institute^24^ as stipulated by the AWERB.

### Lung deposition assay

8-week old female CD1 nude mice (Charles River) were injected intravenously with 1 × 10^5^ of either control (*n*=7) or LOX-depleted (*n*=7) MDA-MB-231 cells in PBS. Randomization to groups was by non-statistical methods and sample number per group was based on past experimental results. Mice were monitored unblinded for up to 50 days post-injection and humanely killed at the end of the experiment, or when animals showed signs of ill health. Lungs were collected post-mortem.

### LOX inhibitor treatment on mouse breast cancer model

MMTV-PyMT^32^ (FVB) female mice were randomized by non-statistical methods to LOX inhibitor treatment groups from day 70 post-birth, when animals had no detectable tumour. Mice were treated daily by oral gavage with 70 mg kg^−1^ CCT365623 (*n*=6) or 70 mg kg^−1^ BAPN (3-aminopropionitrile fumarate, Fisher Scientific) (*n*=4) in vehicle (5% DMSO/2.5% Tween20 in water) or controls with vehicle alone (*n*=6). An alternative group of *n*=7 were given 0.01 g ml^−1^ (1% w/v) BAPN in drinking water or untreated drinking water for *n*=7 additional controls. The water intake of an adult mouse is∼3.64 ml day^−1^ and an adult mouse weighs around 25 g, thus the BAPN therapy dose is calculated as follows: 0.01 × 3.64 ml per 25 g=0.0364, g per 25 g=1,460 mg kg^−1^. Sample number per group was based on past experimental results. Tumour size was determined unblinded by caliper measurements of tumour length, width and depth and volume was calculated as 0.5236 × length × width × depth (mm). In all experiments, mice were humanely killed and mammary tumours and lungs collected when the primary tumours reached ethical size limits or signs of ill health.

### Histology and immunohistochemistry

All mouse tissue samples were fixed in 10% formalin (Sigma) and embedded in paraffin. Samples were sectioned, and hematoxylin and eosin (H&E) stained using standard protocols. H&E stained samples were imaged with a Leica DM4000 upright microscope. To quantify malignant deposits in the lungs following intravenous injection of MDA-MB-231 cells, the proportion of lung parenchyma occupied was assessed and scored as described in Supplementary Table 1. The investigator was blinded to the experimental groups. For spontaneous lung metastasis in MMTV-PyMT animals treated with either water or BAPN, we counted the number of lung metastasis in the lung parenchyma ranked by size and a lung metastasis score was assigned accordingly as described in Supplementary Table 4. The investigator was blinded to the experimental groups. Anti-Mouse MATN2 (R&D, AF3044) was used at 1:100 dilution. Anti-EGFR (Abcam, ab52894) was used at 1:200 dilution to stain sectioned mouse tissue samples. Corresponding fluorescence secondary antibodies (Life Technologies) were then used for staining for confocal imaging of tissue samples.

### LOX inhibitor

The synthetic route of CCT365623 (compound **2** in Fig. 6a) is detailed in Supplementary Methods. For LOX inhibition assays in cell culture, BAPN was dissolved in water and CCT365623 was dissolved in DMSO. Cells were treated at indicated concentrations overnight, with the exception of the MDCK cysts which were treated with LOX inhibitors just before imaging.

### *In vitro* pig skin LOX HRP enzyme activity assay

LOX enzyme was extracted from pig skin^33^. The following procedures were all performed at 4 °C. Pig skins were minced and mixed in PBS (0.09 M Na_2_HPO_4_; 0.01 M NaH_2_PO_4_; 0.15 M NaCl, pH 7.8) using a large food mixer. Samples were centrifuged at 10,000*g* for 20 min. Pellets were collected and washed in PB (PBS without NaCl) and re-suspended in 10 mM CuCl_2_ in PBU (PB with 6 M urea) and stirred over night using a magnetic stirrer. Supernatant was collected next day after centrifugation at 10,000*g* for 20 min. LOX protein was purified from the supernatant using AKTAexplorer 100 (GE) fitted with a XK Column (GE, XK26/100) filed with DEAE Sepharose Fast Flow (GE). Flow rate was set at 2.5 (∼1 ml min^−1^). The XK Column was washed with PB. LOX protein was eluted using PBU.

Pig skin LOX enzyme was diluted according to specific pre-determined activity in assay buffer (100 mM CHES, pH 9, 0.05% (w/v) pluronic F-127, 0.5% (w/v) BSA, 1 mM MgCl_2_, 1 M urea, 100 mM NaCl). Overall, 0.5 μl test compound was added to 40 μl assay buffer+LOX at 100, 30, 10, 3, 1, 0.3, 0.1, 0.03, 0.01 and 0.003 μM (final concentration) in a 96-well black, flat bottomed plate (Corning) and incubated for 20 min at room temperature. The reaction was initiated by addition of 10 μl freshly prepared start mix (20 mM cadaverine dihydrochloride (Sigma), 20 μM Amplex Red (Thermo Fisher Scientific), 1 U ml^−1^ horseradish peroxidase (HRP), final concentration); in assay buffer and the plate was incubated for 45 min at room temperature, protected from light. Fluorescence was measured at 545/585 nm (Ex/Em) using a SpectaMax M5 plate reader and resulting IC_50_ values were calculated using GraphPad Prism software.

### *In vitro* amino oxidase activity assay

The Promega MAO-Glo assay kit was used to assess MAO-A and MAO-B activity. MAO substrate was prepared at 80 μM with the specific MAO-A/B reaction buffer. Overall, 0.5 μl test compound was added to 25 μl MAO substrate at 100, 30, 10, 3, 1, 0.3, 0.1, 0.03, 0.01 and 0.003 μM (final concentration) in a 96-well white, flat bottom plate. Overall, 25 μl of 1:520 dilution of MAO-A enzyme (Promega, V1452) or 25 μl of 1:52 dilution of MAO-B enzyme (Sigma, M7441) in specific MAO-A/B reaction buffer was added to each well (enzyme prepared at 2 × final concentration) and plates were incubated for 1 h at room temperature. Finally, 50 μl per well luciferin detection reagent was added and plates were incubated for 20 min at room temperature, protected from light. Luminescence was measured with an integration time of 500 ms per well using a SpectaMax M5 plate reader and resulting IC_50_ values were calculated using GraphPad Prism software.

The DAO catalytic activity was determined using the Promega ROS Glo assay kit, DAO was purchased from Sigma and cadaverine dihydrochloride was used as the substrate at a concentration of 97.8 mM. The concentrations of the chemical inhibitors were the same as in the MAO assays. Luminescence was measured with an integration time of 500 ms/well using a SpectaMax M5 plate reader and resulting IC_50_ values were calculated using GraphPad Prism software.

### Pharmacokinetic analysis

For pharmacokinetic analyses, CCT365623 or BAPN (50 mg kg^−1^ in 5%DMSO/water, PO *n*=21 or 10 mg kg^−1^ in 5%DMSO/water, IV *n*=24) were administered to mice (6 weeks, female, Balb/C and CD1 nude, Charles River). CCT365623 blood samples were collected at time points from 5 min–8 h post dose from the tail vein using 20 μl heparinized capillaries then pipetted onto Whatman FTA DMPK-B cards (GE Healthcare) and left to dry overnight. BAPN blood samples were collected at the same time points, then the plasma pipetted into cryovials, immediately snap frozen in liquid nitrogen and stored at −80 °C overnight.

CCT365623 and BAPN solutions (10 mM in DMSO) were used to make stock standard curve (SC) and quality control (QC) solutions at appropriate concentrations. Blank blood (for CCT365623) or blank plasma (for BAPN) was spiked with stock solutions to produce a final SC concentration of 10, 50, 100, 500 1,000, 5,000 and 10,000 nM; with final QC concentrations of 250 and 7,500 nM. For CCT365623 SC blood (20 μl) and QC blood (20 μl) was pipetted onto Whatman FTA DMPK-B cards (GE Healthcare) and left to dry overnight. To extract, samples, SC and QC blood spots were punched from Whatman cards with Harris unicore 6 mm punch (GE Healthcare) and placed in a 1 ml 96-well plate. Methanol (200 μl) containing internal standard was added. Samples were vortex mixed for 10 min then centrifuged at 3,700 r,p,m., 4 °C for 5 min. Owing to its small size, a dansyl derivative of BAPN was used in PK analysis^34^. For BAPN derivatization, 10 μl SC samples; 10 μl QC samples; and 10 μl of 1:10 plasma dilution samples; were each mixed with 20 μl dansyl chloride (10 mg ml^−1^ in acetone) and 20 μl sodium bicarbonate (100 mM, pH 11). The samples were incubated for 10 min at 60 °C. 50 μl distilled water was then added before centrifugation at 14,000*g* for 10 min. To extract the derivatized BAPN, a solid phase extraction plate (Supelco Protein precipitation plate) was conditioned under vacuum with 1 ml deionized water and 1 ml methanol. Overall, 85 μl supernatant from the samples was added to the plate and the cartridge washed with 100 μl methanol:water (30:70). Elution was carried out with 200 μl methanol (with formic acid at 2%). The samples were evaporated under nitrogen and then reconstituted in 30 μl methanol:water (30:70). The plate was vortex mixed and centrifuged for 5 min at 3,700 r.p.m. Extracted samples from BAPN and CCT365623 were then diluted for analysis by liquid chromatography mass spectrometry (LC–MS/MS) for the compound concentrations. Non-compartmental analysis was performed on concentration data by computer software WinNonlin v6.3.

### Liver microsome metabolism

A cofactor incubation solution containing 10 mM PBS, 10 mM MgCl_2_, 2 mM NADH, and 2 mM NADPH was prepared. Microsomes (BALB/c, custom batch, Tebu-bio), were added to the cofactor solution as a 1:20 dilution from 0.5 mg ml^−1^ solution. The required compounds were diluted to 10 μM in the cofactor solution containing microsomes. Microsome-incubated samples were assessed at 0, 15 and 30 min. Control samples containing no microsomes and no cofactors were also assessed at 0 and 30 min. Samples were extracted by protein precipitation, and centrifugation for 20 min in a refrigerated centrifuge (4 °C) at 3,700 r.p.m. The supernatant was analysed by LC–MS/MS for the compound concentrations, and % metabolized over time was calculated.

### Statistics

Student's *t*-test was used to calculate significance of differences in all experiments except lung deposit score and lung metastasis score, for which non-parametric Mann–Whitney analysis was used. All tests were two-sided. In Student's *t*-test, a *P* value <0.01 was considered significant. While in the non-parametric Mann–Whitney analysis, a *P* value <0.05 was considered significant. All statistical analysis was performed using Microsoft Excel or GraphPad Prism.

### Data availability

The authors declare that data supporting the findings of this study are available within the article and the Supplementary Information, or available from the authors upon request.

## Additional information

**How to cite this article:** Tang, H *et al*. Lysyl oxidase drives tumour progression by trapping EGF receptors at the cell surface. *Nat. Commun.* **8**, 14909 doi: 10.1038/ncomms14909 (2017).

**Publisher's note:** Springer Nature remains neutral with regard to jurisdictional claims in published maps and institutional affiliations.

## Supplementary Material

Supplementary Information Supplementary Figures, Supplementary Tables and Supplementary Methods
