# Author Correction: Lysyl oxidase drives tumour progression by trapping EGF receptors at the cell surface

**DOI:** 10.1038/s41467-019-11220-x

**Published:** 2019-07-18

**Authors:** HaoRan Tang, Leo Leung, Grazia Saturno, Amaya Viros, Duncan Smith, Gianpiero Di Leva, Eamonn Morrison, Dan Niculescu-Duvaz, Filipa Lopes, Louise Johnson, Nathalie Dhomen, Caroline Springer, Richard Marais

**Affiliations:** 1 Molecular Oncology Group, Cancer Research UK Manchester Institute, University of Manchester, Manchester, M20 4BX UK; 2 Gene and Oncogene Targeting Team, CRUK Cancer Therapeutics Unit, The Institute of Cancer Research, London, SM2 5NG UK; 3 Biological Mass Spectrometry Unit, Cancer Research UK Manchester Institute, University of Manchester, Manchester, M20 4BX UK

Correction to: *Nature Communications* 10.1038/ncomms14909, published online 18 April 2017.

This Article contains errors in Figs. 1, 2, 4, Supplementary Fig. 1c and Supplementary Fig. 3f, for which we apologise.

In Figs. 1c, 2c, 4j and Supplementary Fig. 1, samples from multiple biological replicates of each condition were run and probed; however, the blot images were assembled incorrectly such that different replicates were inadvertently selected as representative images for a subset of the antibodies used. The following blots depict a different biological replicate compared with the other blots within each experiment:

In Fig. 1c, the blot depicting surface EGFR levels in MDA-MB-231 cells and the blots depicting total EGFR and GAPDH levels in U87 cells.

In Fig. 2c, the blots depicting pAKT, total AKT and GAPDH levels.

In Fig. 4j, the blot depicting pY1068EGFR levels.

In Supplementary Fig. 1, the blots depicting pY1068EGFR levels in both MDA-MB-231 cells and U87 cells.

The correct versions of Figs. 1c and 2c, 4j and Supplementary Fig. 1c are shown below as Figs. 1–4 respectively.

**Figure 1:**
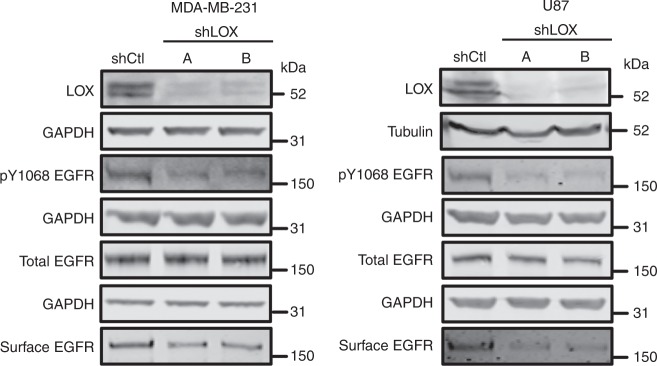
Fig. 1

**Figure 2:**
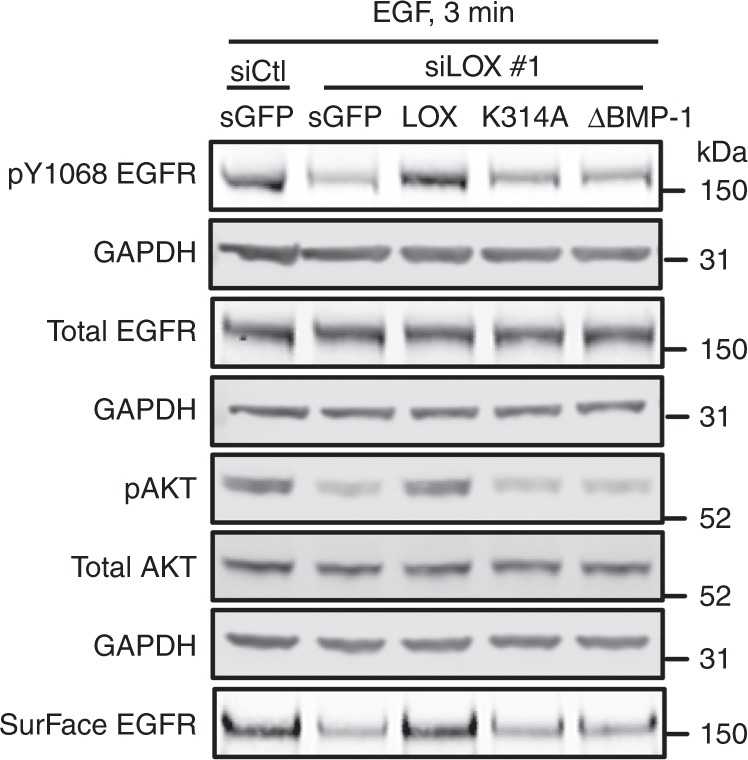
Fig. 2

**Figure 3:**
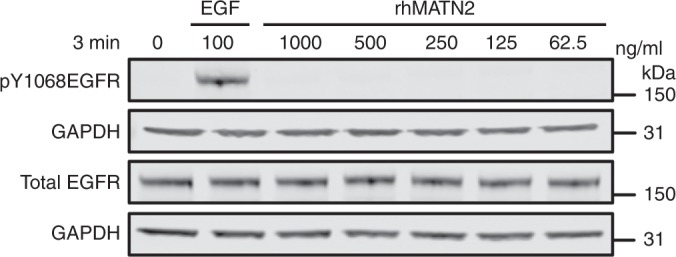
Fig. 3

**Figure 4:**
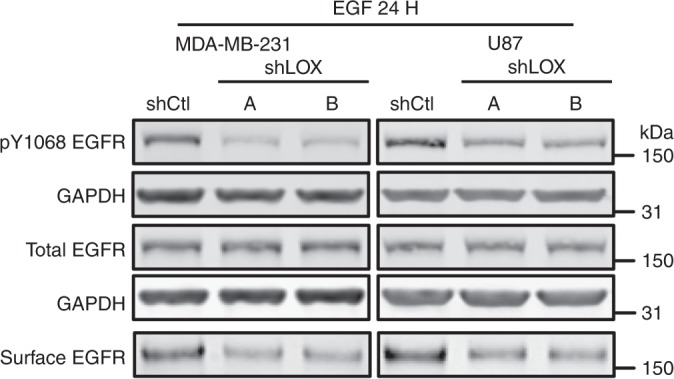
Fig. 4

Furthermore, in Supplementary Fig. 3f, the blots probed with MATN2 and LOX antibodies were derived from samples run on separate gels, however only the GAPDH loading control for the MATN2 blot is depicted. The correct version of Supplementary Fig. 3f, with GAPDH loading controls for both the MATN2 and LOX blots, is shown below as Fig. 5.

**Figure 5:**
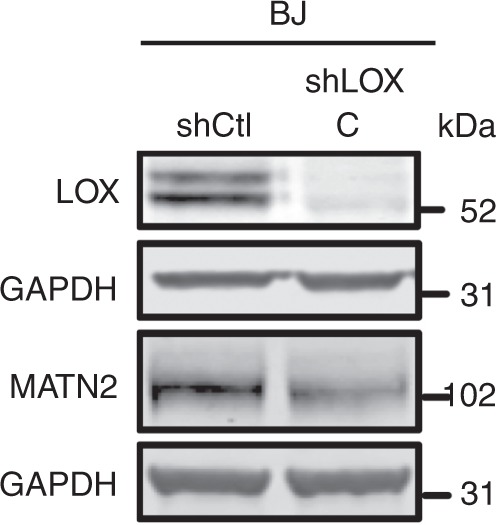
Fig. 5

These errors do not alter the original conclusions of the study. The error has not been corrected in the PDF or HTML versions of the Article.

