# Supplementary material for: Lysyl oxidase drives tumour progression by trapping EGF receptors at the cell surface
Source: Nat Commun. 2017 Apr 18;8:14909. doi: 10.1038/ncomms14909 (PMC5399287; doi:10.1038/ncomms14909)
Supplement: Supplementary Information — Supplementary Figures, Supplementary Tables and Supplementary Methods [file ncomms14909-s1.pdf]

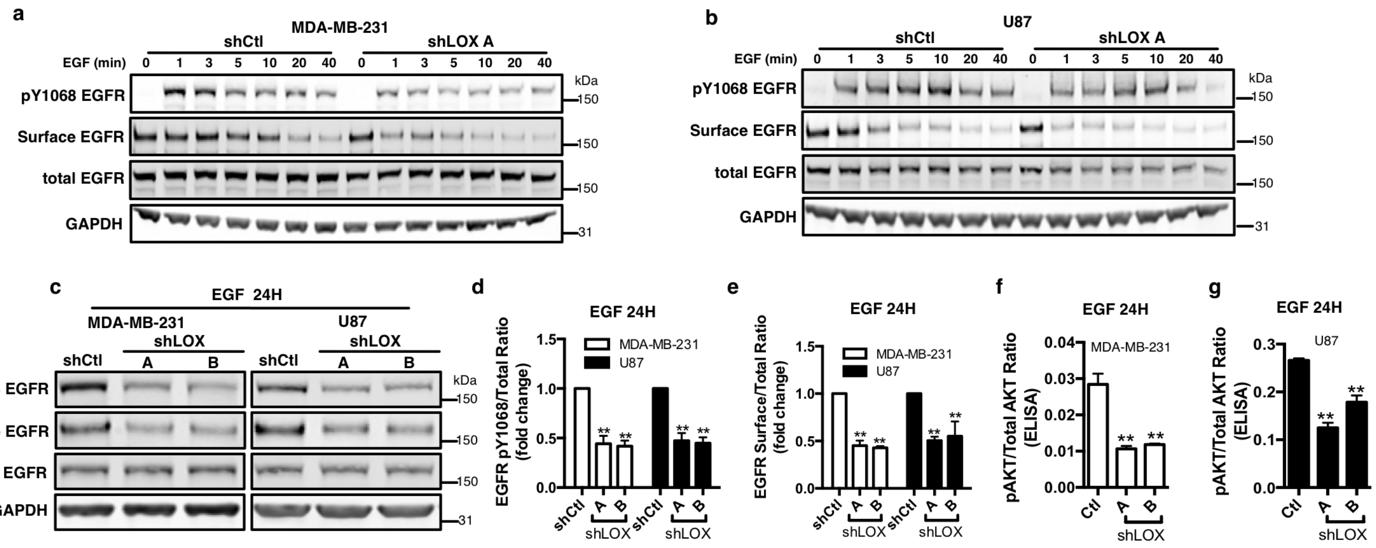

Supplementary Figure 1, LOX controls surface EGFR retention and AKT activation.

(a, b) Western blots of pY1068 EGFR, surface EGFR, total EGFR and GAPDH in EGF-stimulated control (shCtl) or LOX depleted (shLOX A) MDA-MB-231 and U87 cells. (c) Western blots of pY1068 EGFR, surface EGFR, total EGFR and GAPDH in control (shCtl) or LOX depleted (shLOX A,B) MDA-MB-231 and U87 cells cultured under EGF only condition for 24 hours. (d) Quantification of EGFR activation, and (e) surface EGFR level in MDA-MB-231 and U87 cells from c. (f,g) AKT activation in MDA-MB-231 cells and in U87 cells under EGF only condition for 24 hours. All data are represented as Mean ± SD from 3 independent experiments. **p<0.01, Student's t-test.

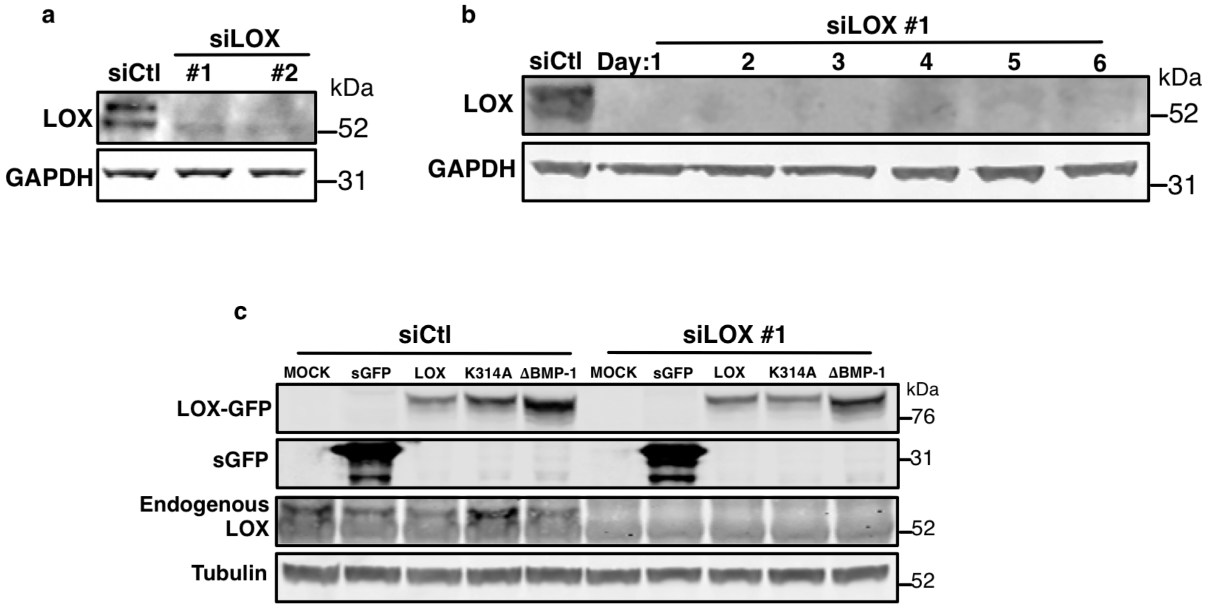

Supplementary Figure 2, LOX RNA interference

(a) Western blot showing LOX and GAPDH in control (siCtl) or LOX depleted (siLOX #1, siLOX #2) MDA-MB-231 cells. (b) Time course (days) showing LOX and GAPDH in LOX depleted (siLOX #1) MDA-MB-231 cells. (c) Western blot showing expression of transfected secreted GFP (sGFP), LOX-GFP (LOX), LOX^{K314A}-GFP (K314A), LOX$^{\Delta BMP-1}$-GFP (ΔBMP-1), endogenous LOX and tubulin in control (siCtl) or LOX depleted (siLOX #1) MDA-MB-231 cells. MOCK: transfection reagents only.

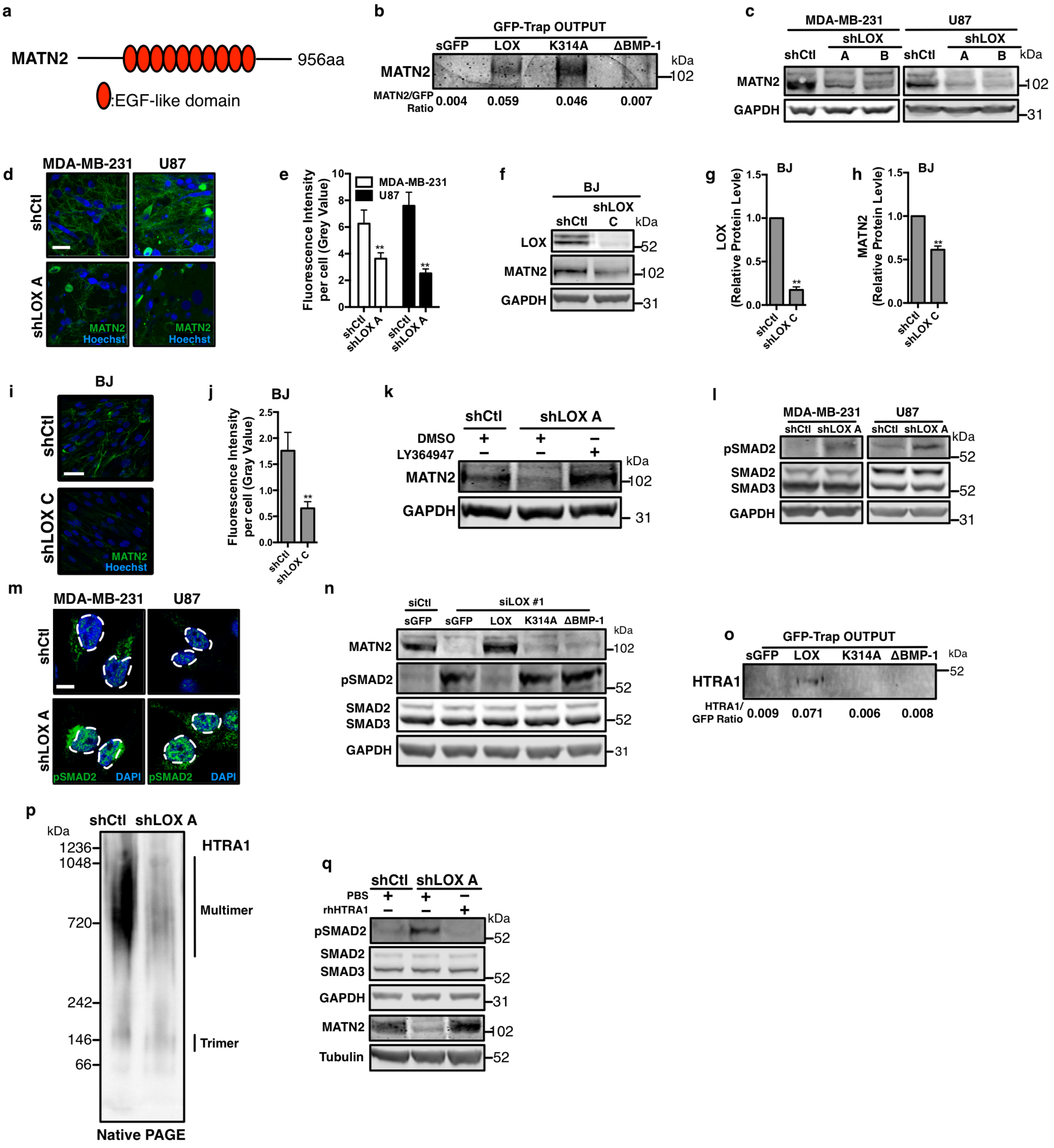

Supplementary Figure 3, LOX regulates MATN2 and HTRA1

(a) Schematic showing 10 EGF-like domains in matrilin2 (MATN2). (b) Western blot showing MATN2 binding to LOX-GFP (LOX), LOX^{K314A}-GFP (K314A). MATN2/GFP ratio indicates binding strength. (c) Western blots showing MATN2 and GAPDH in control (shCtl) or LOX depleted (shLOX A, B) MDA-MB-231 and U87 cells. (d) Confocal photomicrographs of

extracellular MATN2 in control (shCtl) or LOX depleted (shLOX A) MDA-MB-231 and U87 cells in collagen gels. Scale bar: 40µm. (e) Quantification of extracellular MATN2 in d. All data are represented as Mean ± SD from 15 fields of views per condition. **p<0.01, Student's t-test. (f) Western blots showing LOX, MATN2 and GAPDH in control (shCtl) or LOX depleted (shLOX C) BJ fibroblasts. (g,h) Quantification of LOX and MATN2 protein expression from experiments in f. All data are represented as Mean ± SD from 3 independent experiments. **p<0.01, Student's t-test. (i) Confocal photomicrographs of extracellular MATN2 in cell derived matrix (CDM) of control (shCtl) or LOX depleted (shLOX C) BJ fibroblasts. Scale bar: 40µm. (j) Quantification of extracellular MATN2 in i. Data are represented as Mean ± SD from 15 fields of views per condition. **p<0.01, Student's t-test. (k) Western blot showing MATN2 in DMSO or LY364947 treated control (shCtl) or LOX depleted (shLOX A) MDA-MB-231 cells. (l) Western blots showing pSMAD2, SMAD2/3 and GAPDH in control (shCtl) or LOX depleted (shLOX A) MDA-MB-231 and U87 cells. (m) Confocal photomicrographs of nuclear phospho-SMAD2 (pSMAD2) in control (shCtl) or LOX depleted (shLOX A) MDA-MB-231 and U87 cells. Scale bar: 10µm. (n) Western blot for MATN2, pSMAD2, SMAD2/3, and GAPDH in control (siCtl) or LOX depleted (siLOX #1) MDA-MB-231 cells expressing secreted GFP (sGFP), LOX-GFP (LOX), LOX^{K314A}-GFP (K314A) or LOX$^{\Delta BMP\text{-}1}$-GFP (ΔBMP-1). (o) Western blot showing HTRA1 binding to LOX-GFP (LOX). HTRA1/GFP ratio indicates binding strength. (p) NativePAGE showing extracellular HTRA1 multimer and trimer in control (shCtl) or LOX depleted (shLOX A) MDA-MB-231 cell culture medium. (q) Western blot showing pSMAD2, SMAD2/3, GAPDH, MATN2 and Tubulin in PBS or rhHTRA1 treated control (shCtl) or LOX depleted (shLOX A) MDA-MB-231 cells.

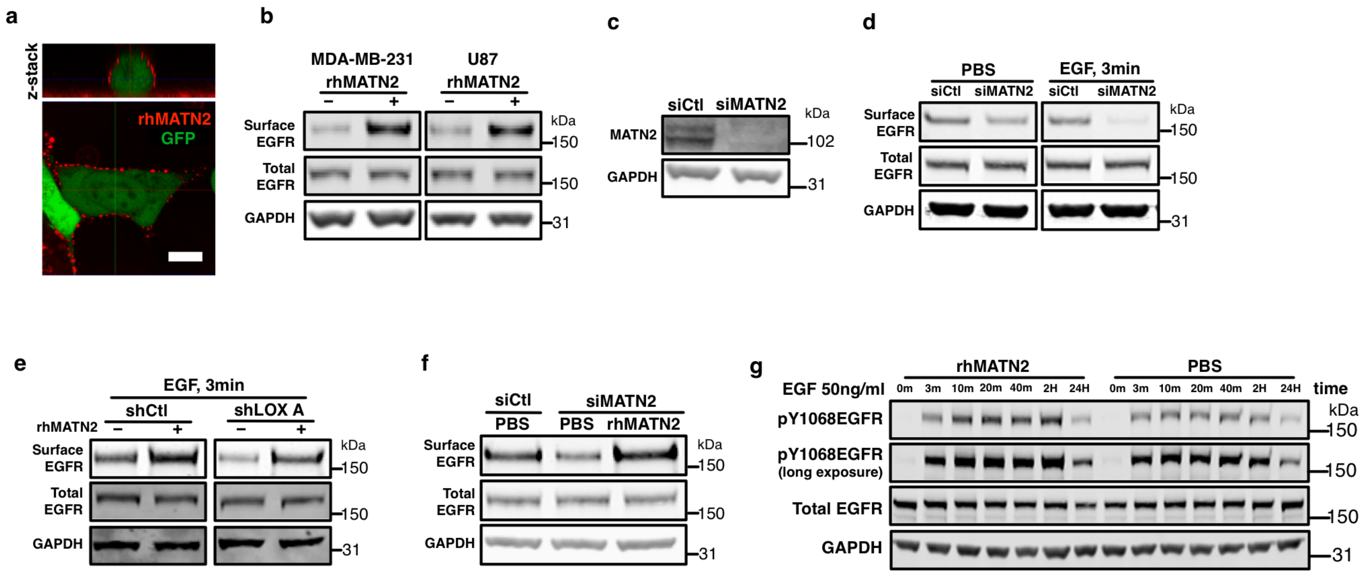

Supplementary Figure 4, MATN2 upregulates cell surface EGFR.

(a) Confocal photomicrographs of rhMATN2 (Red) binding to the cell surface of a GFP expressing MDA-MB-231 cell (Green). Scale bar: 10μm. (b) Western blots of surface EGFR, total EGFR and GAPDH in rhMATN2 treated (500ng/ml) MDA-MB-231 and U87 cells. (c) Western blots showing MATN2 and GAPDH in control (shCtl) or MATN2 depleted (siMATN2) MDA-MB-231 cells. (d) Western blots showing surface EGFR, total EGFR and GAPDH in PBS or EGF treated (3 min) control (siCtl) or MATN2 depleted (siMATN2) MDA-MB-231 cells. (e) Western blots showing surface EGFR in rhMATN2 treated control (shCtl) or LOX depleted (shLOX A) MDA-MB-231 cells under 3min EGF stimulation. (f) Western blots showing surface EGFR, total EGFR and GAPDH in PBS or rhMATN2 treated control (siCtl) or MATN2 depleted (siMATN2) MDA-MB-231 cells. (g) Western blots showing pY1068EGFR, total EGFR and GAPDH in EGF (50ng/ml) or EGF(50ng/ml) and rhMATN2 (500ng/ml) treated MDA-MB-231 cells at indicated time points.

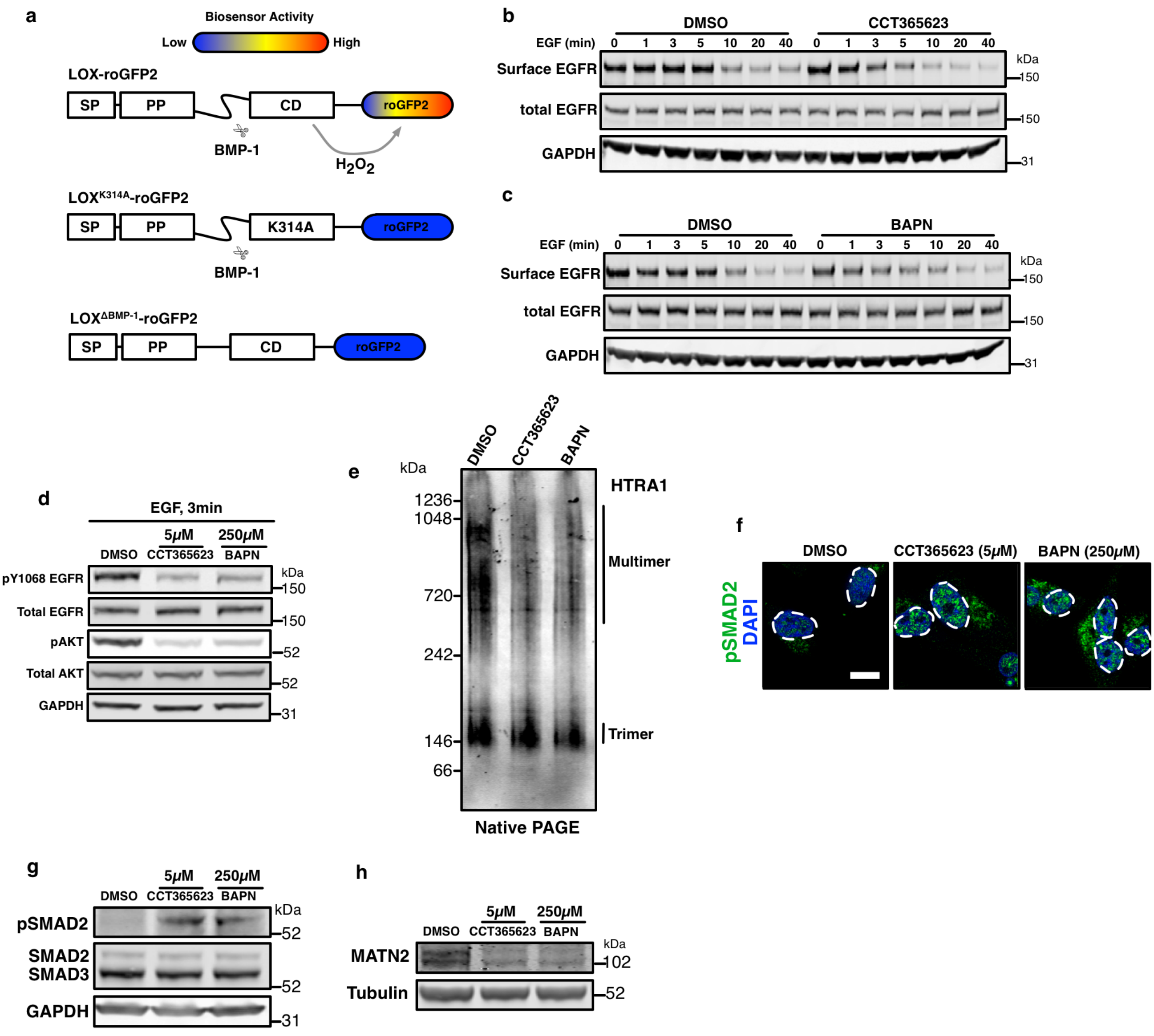

Supplementary Figure 5, LOX chemical inhibition controls surface EGFR via MATN2

(a) Schematic illustrating the LOX biosensor (LOX-roGFP2) mechanism used in this study. SP, signal peptide; PP, pro-peptide; CD, catalytic domain; LOX, wild type enzyme; K314A, catalytically inactive LOX mutant; ΔBMP-1; BMP-1 cleavage site deleted LOX mutant. (b,c) Western blots showing surface EGFR, total EGFR and GAPDH in EGF-stimulated DMSO, CCT365623, or BAPN treated MDA-MB-231 cells. (d) Western blots for pY1068 EGFR, total EGFR, pAKT, total AKT and GAPDH in EGF (3 min) stimulated, DMSO, CCT365623 or BAPN treated MDA-MD-231 cells. (e) Western blot from Native PAGE showing reduced HTRA1 multimer in

DMSO, CCT365623, or BAPN treated MDA-MB-213 cell culture media. (f) Confocal photomicrographs showing nuclear phospho-SMAD2 (pSMAD2) in DMSO, CCT365623 or BAPN treated MDA-MB-231 cells. Scale bar, 10μm. (g) Western blots showing pSMAD2, SMAD2/3, and GAPDH in DMSO, CCT365623, or BAPN treated MDA-MB-231 cells. (h) Western blots showing MATN2 and tubulin in DMSO, CCT365623, or BAPN treated MDA-MB-231 cells.

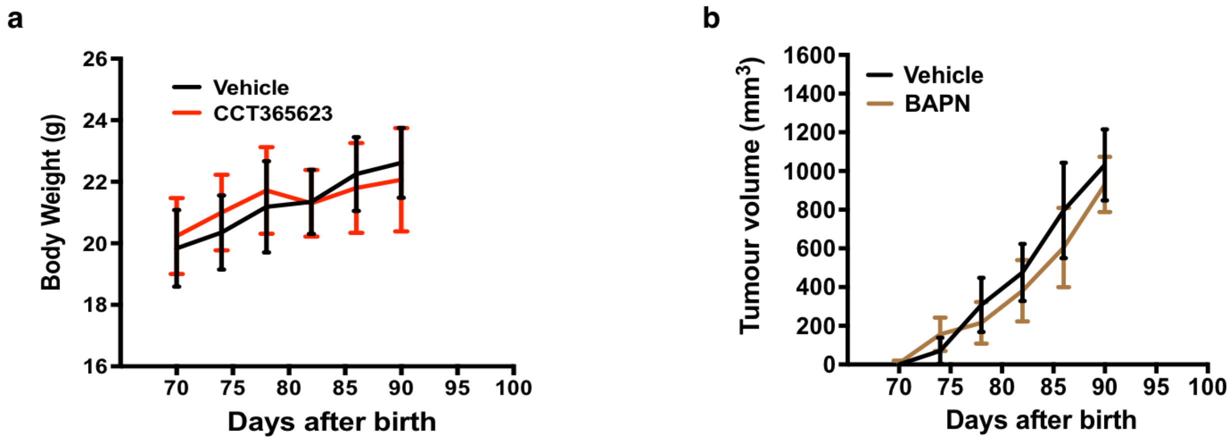

Supplementary Figure 6, (a) Mouse body weight in Vehicle or CCT365623 treated MMTV-PyMT mice. Data are represented as Mean ± SD from 6 mice in Vehicle group, and 6 mice in CCT365623 group. (b) Tumour growth in Vehicle or BAPN treated MMTV-PyMT mice. Data are represented as Mean ± SD from 6 mice in Vehicle group, and 4 mice in BAPN group.

Fig.1c MDA-MB-231 LOX

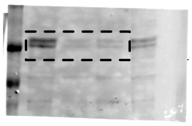

Fig.1c MDA-MB-231 pY1068 EGFR

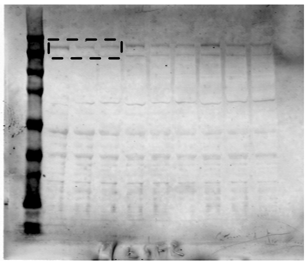

Fig.1c MDA-MB-231 Surface EGFR

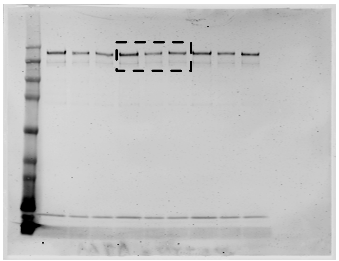

Fig.1c MDA-MB-231 Total EGFR

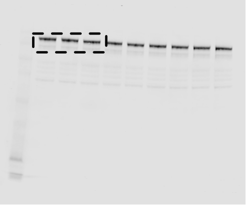

Fig.1c MDA-MB-231 GAPDH

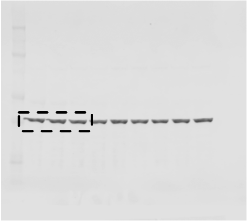

Fig.1c U87 LOX

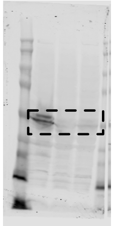

Fig.1c U87 pY1068 EGFR

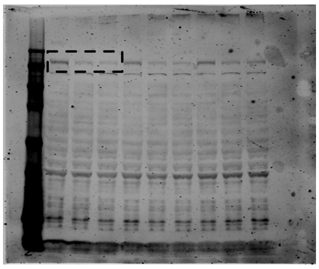

Fig.1c U87 surface EGFR

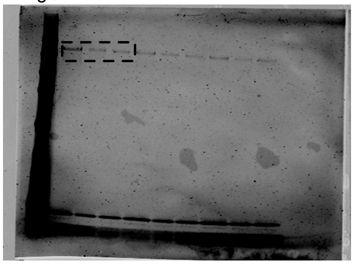

Fig.1c U87 Total EGFR

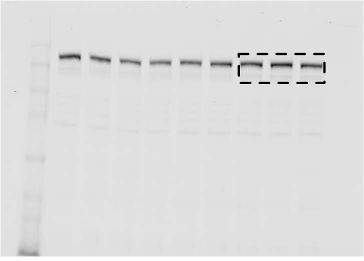

Fig.1c U87 GAPDH

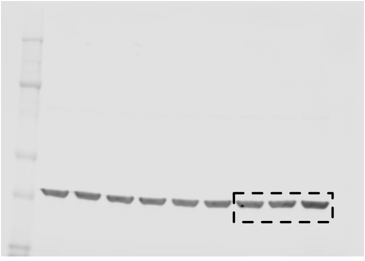

Fig.2c pY1068 EGFR

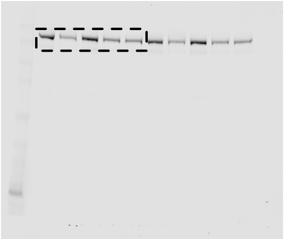

Fig.2c Surface EGFR

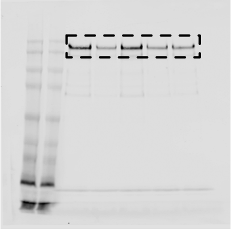

Fig.2c Total EGFR

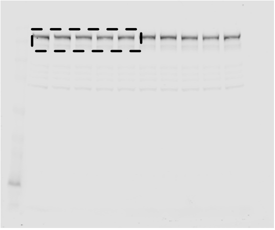

Fig.2c pAKT

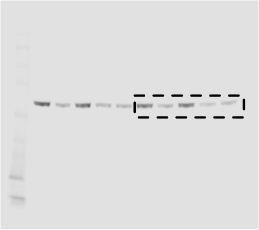

Fig.2c Total Akt

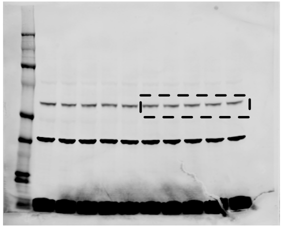

Fig.2c GAPDH

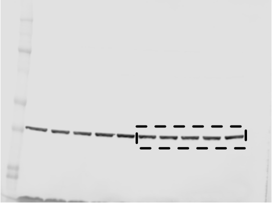

Fig.4i pY1068EGFR

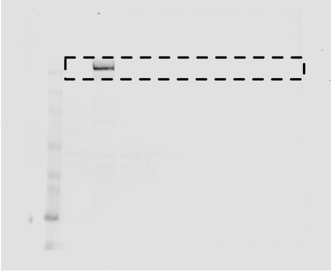

Fig.4i Total EGFR

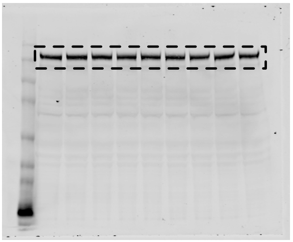

Fig.4i GAPDH

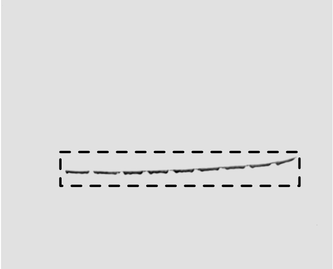

Fig.4j pY1068 EGFR

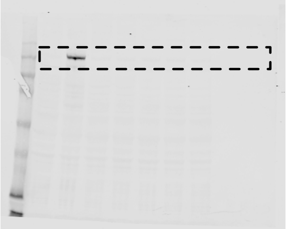

Fig.4j Total EGFR

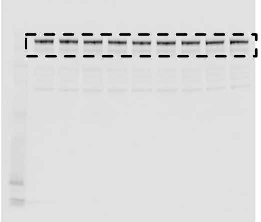

Fig.4j GAPDH

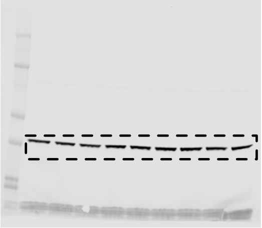

Supplementary Fig. 1a
pY1068 EGFR

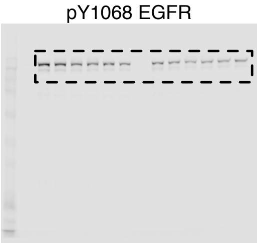

Supplementary Fig. 1a
Surface EGFR

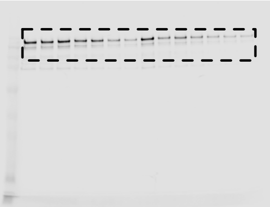

Supplementary Fig. 1a
Total EGFR

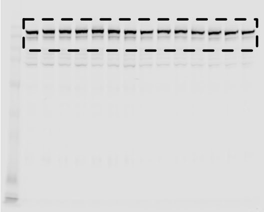

Supplementary Fig. 1a
GAPDH

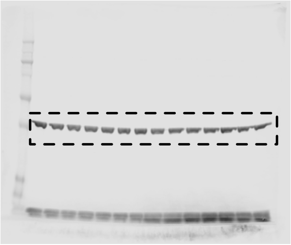

Supplementary Fig. 1b
pY1068 EGFR

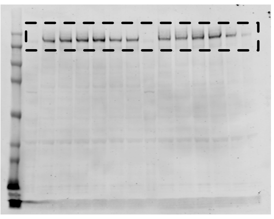

Supplementary Fig. 1b
Surface EGFR

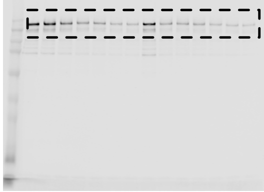

Supplementary Fig. 1b
Total EGFR

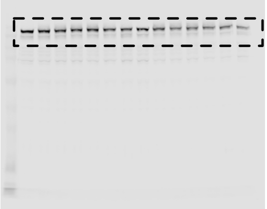

Supplementary Fig. 1b
GAPDH

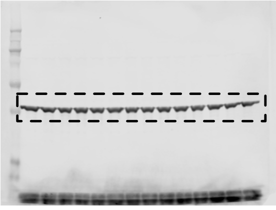

Supplementary Fig. 1c
MDA-MB-231 pY1068 EGFR

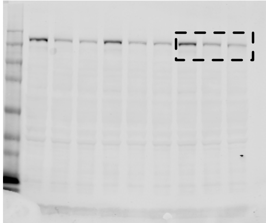

Supplementary Fig. 1c
MDA-MB-231 surface EGFR

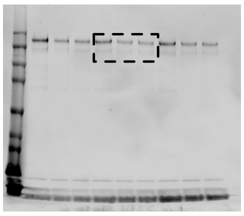

Supplementary Fig. 1c
MDA-MB-231 Total EGFR

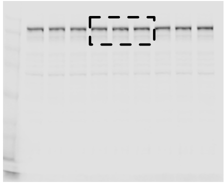

Supplementary Fig. 1c
MDA-MB-231 GAPDH

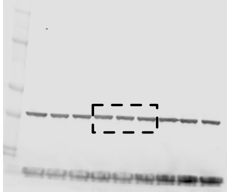

Supplementary Fig. 1c
U87 pY1068 EGFR

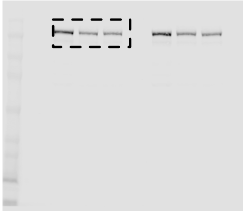

Supplementary Fig. 1c
U87 surface EGFR

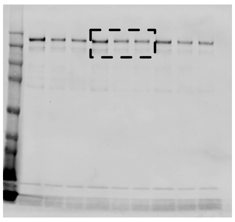

Supplementary Fig. 1c
U87 Total EGFR

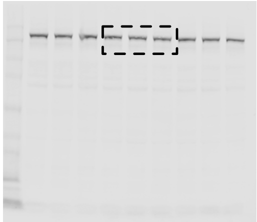

Supplementary Fig. 1c
U87 GAPDH

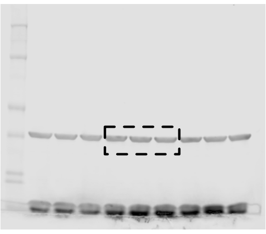

Supplementary Fig. 2a
LOX

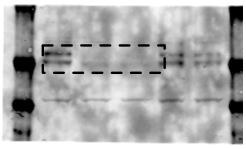

Supplementary Fig. 2a
GAPDH

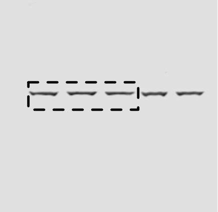

Supplementary Fig. 2b
LOX

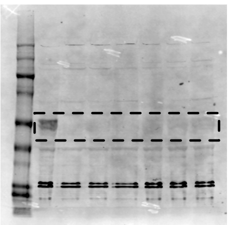

Supplementary Fig. 2b
GAPDH

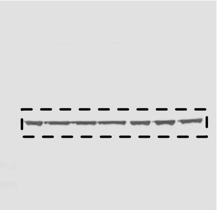

Supplementary Fig. 2c
LOX-GFP, sGFP

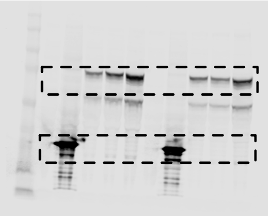

Supplementary Fig. 2c
Endogenous LOX

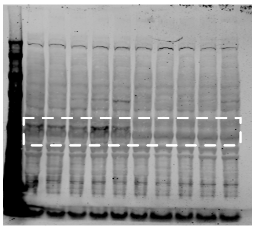

Supplementary Fig. 2c
Tubulin

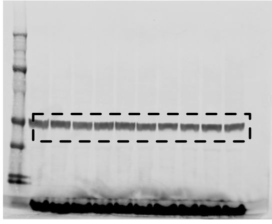

Supplementary Fig. 3b
MATN2

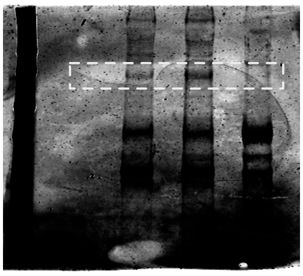

Supplementary Fig. 3c
MDA-MB-231 MATN2

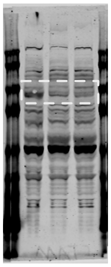

Supplementary Fig. 3c
MDA-MB-231 GAPDH

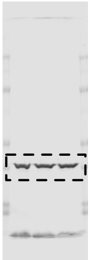

Supplementary Fig. 3c
U87 MATN2

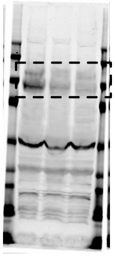

Supplementary Fig. 3c
U87 GAPDH

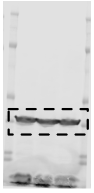

Supplementary Fig. 3f
LOX

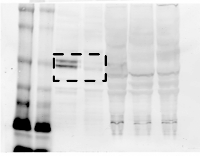

Supplementary Fig. 3f
MATN2

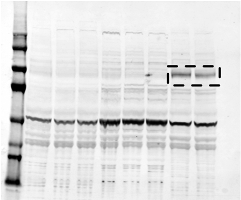

Supplementary Fig. 3f
GAPDH

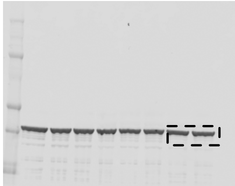

Supplementary Fig. 3k
MATN2

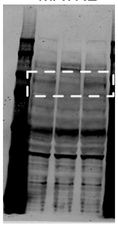

Supplementary Fig. 3k
GAPDH

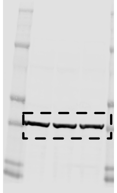

Supplementary Fig. 3l
MDA-MB-231 pSMAD2

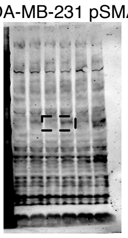

Supplementary Fig. 3l
MDA-MB-231 SMAD2/3

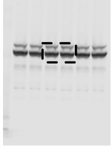

Supplementary Fig. 3l
MDA-MB-231 GAPDH

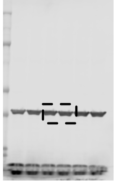

Supplementary Fig. 3l
U87 pSMAD2

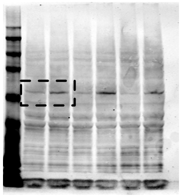

Supplementary Fig. 3l
U87 SMAD2/3

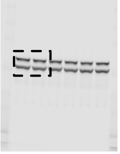

Supplementary Fig. 3l
U87 GAPDH

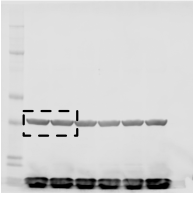

Supplementary Fig. 3n
MATN2

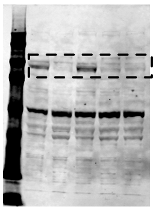

Supplementary Fig. 3n
pSMAD2

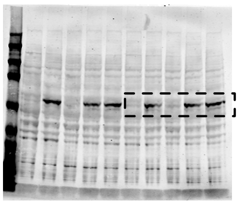

Supplementary Fig. 3n
SMAD2/3

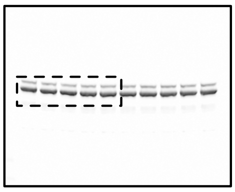

Supplementary Fig. 3n
GAPDH

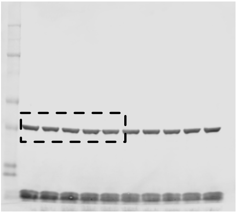

Supplementary Fig. 3o
HTRA1

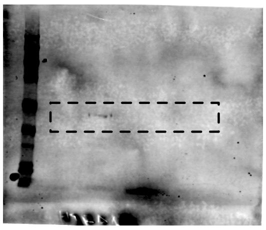

Supplementary Fig. 3q
pSMAD2

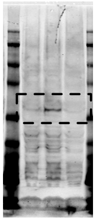

Supplementary Fig. 3q
pSMAD2

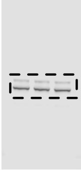

Supplementary Fig. 3q
GAPDH

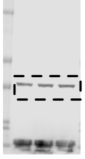

Supplementary Fig. 3q
MATN2

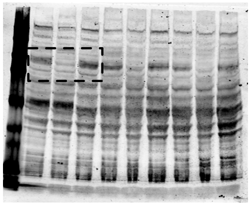

Supplementary Fig. 3q
Tubulin

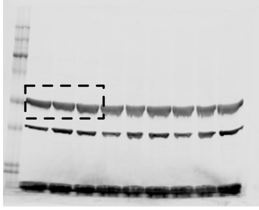

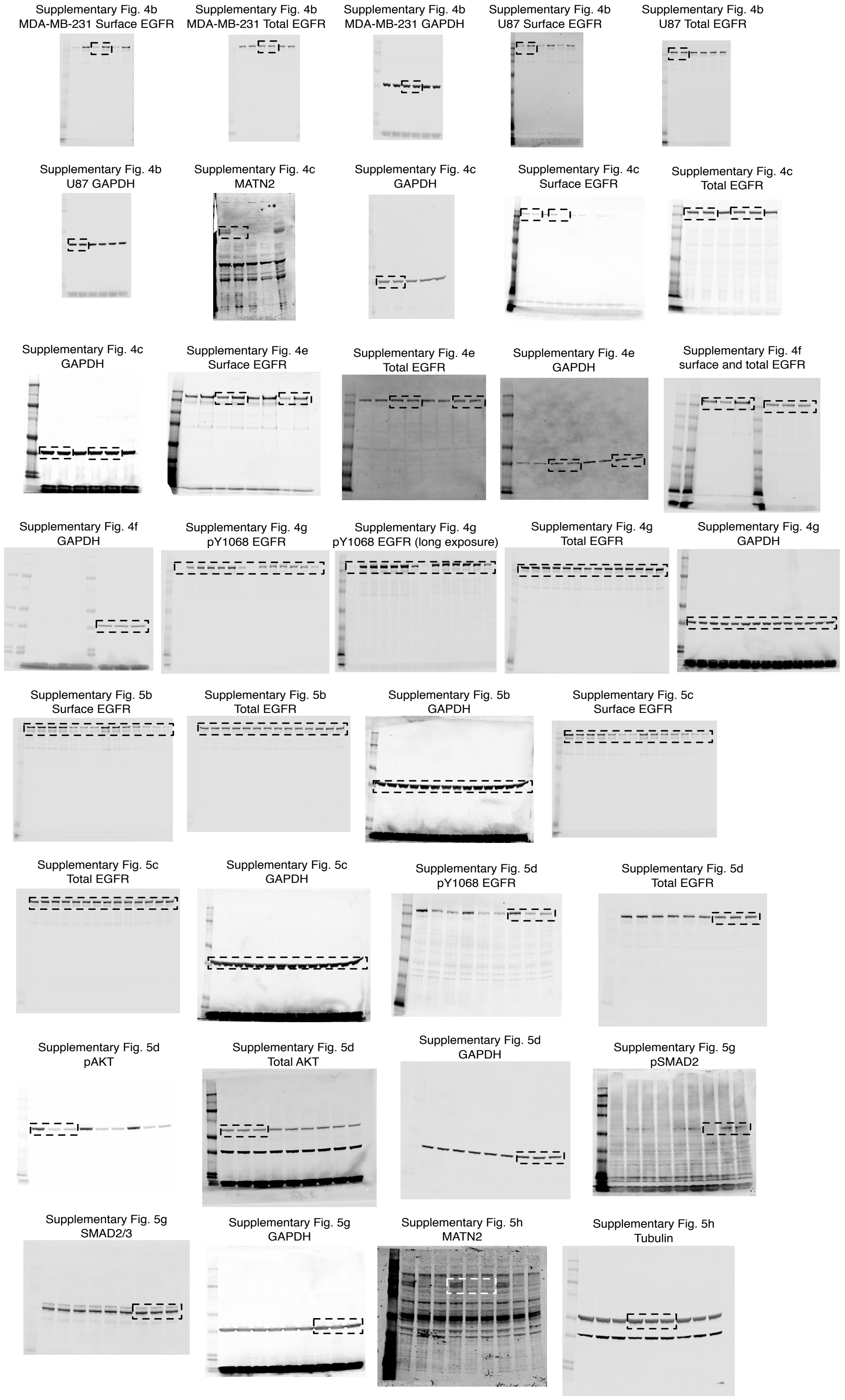

Supplementary Figure 7, Full scans of blots in indicated Figures and Supplementary Figures.

	Mice	Proportion of lung parenchyma occupied by MDA-MB-231 Deposition	Score
**shCtl**	1	50%-75%	3
	2	>75%	4
	3	1-25%	1
	4	25-50%	2
	5	>75%	4
	6	>75%	4
	7	1-25%	1
**shLOX A**	8	25-50%	2
	9	1-25%	1
	10	1-25%	1
	11	0	0
	12	25-50%	2
	13	25-50%	2
	14	<1%, Micromets	0
**Scoring system**		*<1%*	*0*
		1%-25%	*1*
		25-50%	*2*
		50%-75%	*3*
		>75%	*4*

Supplementary Table 1, LOX affects MDA-MB-231 tumour growth in the lungs. Quantification of lung deposits in mice following tail vein injection with control (shCtl) or LOX depleted (shLOX A) MDA-MB-231 cells.

	LOX IC_{50}	DAO IC_{50}	MAO-A IC_{50}	MAO-B IC_{50}	hERG IC_{50}	MLM
**CCT365623**	0.89μM	>1mM	333μM	>1mM	>20μM	65%
**BAPN**	14.7μM	204μM	>1mM	>1mM	>20μM	70%

Supplementary Table 2, Characterisation of CCT365623 and BAPN. The table shows the IC_{50} for CCT365623 and BAPN against LOX, diamine oxidase (DAO), monoamine oxidase A and B (MAO-A, MAO-B) and the potassium ion channel hERG. It also shows CCT365623 and BAPN stability in mouse liver microsomes (MLM) *in vitro* (% remaining after 30 minutes incubation).

		PK parameters								Therapy parameters		
	MW (g/mol)	PK Dose (mg/kg)	PK Molar dose (mmol/kg)	C_{max} IV (µM)	C_{max} PO (µM)	F%	$T_{1/2}$ PO (h)	Cl IV (mL/min/kg)	AUClast PO PK (µM.h)	Therapy dose (PO, qd) mg/kg	Therapy molar dose (PO, qd) mmol/kg	AUClast PO extrapolated at therapy dose (µM.h)
**CCT365623**	407	PO - 50<br>IV - 10	PO - 0.12<br>IV - 0.024	17	17	45	0.6	49	15	70	0.17	21
**BAPN**	128	PO - 50<br>IV - 10	PO - 0.39<br>IV - 0.078	132	192	41	0.6	25	195	1460	11.4	5694

Supplementary Table 3. *In Vivo* PK and Therapy Parameters for CCT365623 and BAPN. The table shows the PK parameters for CCT365623 and BAPN following a single PO dose at 50 mg/kg compared to a single IV injection at 10mg/kg respectively. Cmax: maximum concentration. F%: oral bioavailability. $T_{1/2}$: elimination half life. Cl: clearance from plasma. AUClast.: area under the concentration time curve; and the therapy parameters for CCT365623 and BAPN following a single PO dose at 70 mg/kg per day for CCT365623 and an estimated daily dose of 1460 mg/kg for BAPN extrapolated from consumption of compound administered in the drinking water (1% w/v).

<table>
<tr><th></th><th></th><th colspan="3">Number of Metastasis</th><th></th></tr>
<tr><th></th><th>Mice</th><th>Small</th><th>Medium</th><th>Large</th><th>Total Score</th></tr>
<tr><td rowspan="7">Water</td><td>1</td><td>1</td><td>1</td><td>0</td><td>3</td></tr>
<tr><td>2</td><td>12</td><td>12</td><td>12</td><td>72</td></tr>
<tr><td>3</td><td>3</td><td>3</td><td>1</td><td>12</td></tr>
<tr><td>4</td><td>0</td><td>1</td><td>0</td><td>2</td></tr>
<tr><td>5</td><td>0</td><td>1</td><td>0</td><td>2</td></tr>
<tr><td>6</td><td>10</td><td>1</td><td>2</td><td>18</td></tr>
<tr><td>7</td><td>1</td><td>0</td><td>0</td><td>1</td></tr>
<tr><td rowspan="7">BAPN</td><td>8</td><td>0</td><td>0</td><td>0</td><td>0</td></tr>
<tr><td>9</td><td>0</td><td>0</td><td>0</td><td>0</td></tr>
<tr><td>10</td><td>1</td><td>0</td><td>0</td><td>1</td></tr>
<tr><td>11</td><td>1</td><td>0</td><td>0</td><td>1</td></tr>
<tr><td>12</td><td>0</td><td>0</td><td>0</td><td>0</td></tr>
<tr><td>13</td><td>1</td><td>0</td><td>0</td><td>1</td></tr>
<tr><td>14</td><td>0</td><td>0</td><td>0</td><td>0</td></tr>
<tr><td rowspan="6">Vehicle</td><td>1</td><td>7</td><td>0</td><td>5</td><td>22</td></tr>
<tr><td>2</td><td>2</td><td>1</td><td>4</td><td>16</td></tr>
<tr><td>3</td><td>7</td><td>4</td><td>1</td><td>18</td></tr>
<tr><td>4</td><td>1</td><td>2</td><td>9</td><td>32</td></tr>
<tr><td>5</td><td>0</td><td>4</td><td>15</td><td>53</td></tr>
<tr><td>6</td><td>1</td><td>4</td><td>0</td><td>9</td></tr>
<tr><td rowspan="6">CCT365623</td><td>7</td><td>0</td><td>0</td><td>0</td><td>0</td></tr>
<tr><td>8</td><td>0</td><td>0</td><td>0</td><td>0</td></tr>
<tr><td>9</td><td>0</td><td>1</td><td>0</td><td>2</td></tr>
<tr><td>10</td><td>0</td><td>1</td><td>0</td><td>2</td></tr>
<tr><td>11</td><td>0</td><td>1</td><td>0</td><td>2</td></tr>
<tr><td>12</td><td>1</td><td>1</td><td>0</td><td>3</td></tr>
<tr><td rowspan="5" colspan="3">Scoring system</td><td></td><td>Size</td><td>Score</td></tr>
<tr><td>None</td><td>0</td><td>0</td></tr>
<tr><td>Small</td><td><100μm</td><td>1</td></tr>
<tr><td>Medium</td><td>100~200μm</td><td>2</td></tr>
<tr><td>Large</td><td>>200μm</td><td>3</td></tr>
</table>

Supplementary Table 4. Quantification of lung metastases in MMTV-PyMT mice. The table shows the quantification of lung metastases in water or BAPN treated, and vehicle or CCT365623 treated MMTV-PyMT mice. The score was calculated using the following formula (number of small metastasis x 1)+(number of medium metastasis x 2) + (number of large metastasis x 3) for each individual animal.

Supplementary Methods

Synthesis of CCT365623

CCT365623 is compound **2** in Fig.6a. It is referred as **2** in the following methods.

Commercial building blocks **3** and **7**, reagents and solvents for reactions were reagent grade and used as purchased. Chromatography solvents were HPLC grade and were used without further purification. Thin layer chromatography (TLC) analysis was performed using silica gel 60 F-254 thin layer plates. Flash column chromatography was performed using columns pre-packed with 40-63 μm silica. LCMS and HRMS analyses were performed on a HPLC system with diode array detector operating at 254 nm, fitted with a reverse-phase 50×4.6mm column at a temperature of 22 °C, connected to a Time of Flight (ToF) mass spectrometer (ESI). ^{1}H and ^{13}C NMR spectra were recorded on a Bruker Avance 500 MHz spectrometer using an internal deuterium lock. NMR data is given as follows: chemical shift (δ) in ppm, multiplicity, coupling constants (*J*) given in Hz and integration.

(5-((5-(Methylsulfonyl)-[1,1'-biphenyl]-3-yl)sulfonyl)thiophen-2-yl)methanamine (2)

Synthesis route for 3-bromo-5-(methylthio)benzenethiol intermediate ***6***

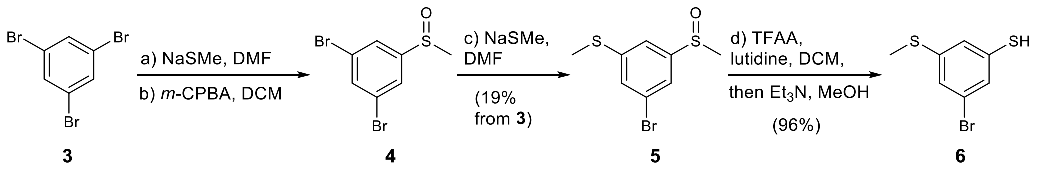

a) NaSMe (21% aq.; 50.8 mL, 152 mmol) was added to a solution of 1,3,5-tribromobenzene **3** (40.0 g, 127 mmol) in DMF (508 mL), and the

reaction was stirred at 100 °C for 16 h. After cooling to rt, EtOAc (1.5 L) was added. The organic phase was washed with H_2O (3 × 1.0 L), brine (1.0 L), dried over $MgSO_4$, filtered and the solvent was removed under reduced pressure. The crude sulfide was used in the subsequent transformation immediately.

b) The intermediate sulfide was dissolved in DCM (400 mL) and cooled to 0 °C. A solution of *m*-CPBA (34.2 g, 152 mmol) in DCM (234 mL) was slowly added and the mixture was stirred at 0 °C for 1 h before allowing to warm to rt over 1 h. EtOAc (800 mL) was added. The organic phase was washed with sat. $NaHCO_3$ (3 × 500 mL), dried over $MgSO_4$, filtered and the solvent was removed under reduced pressure. The crude was recrystallised from DCM/cyclohexane to give 22.3 g of beige crystals containing a 4.5:1 mixture of the desired 1,3-dibromo-5-(methylsulfinyl)benzene **4** and the by-product (5-bromo-1,3-phenylene)bis(methylsulfane). Data for pure **4** - mp: 121–124 °C. **^{1}H NMR** (500 MHz, Chloroform-*d*) δ 7.75 (t, *J* = 1.7 Hz, 1H), 7.67 (d, *J* = 1.7 Hz, 2H), 2.73 (s, 3H). **^{13}C NMR** (126 MHz, Chloroform-*d*) δ 149.76, 136.51, 125.23, 124.02, 44.14. **LCMS (ESI)** *m/z* 297/299/301 $[M+H]^+$.

c) NaSMe (21% aq.; 27.5 mL, 82.3 mmol) was added to a solution of sulfoxide **4** (22.3 g, 74.8 mmol) in DMF (374 mL), and the reaction was stirred at 40 °C for 6 h. After cooling to rt, EtOAc (400 mL) was added. The organic phase was washed with 1:1 H_2O/brine (3 × 400 mL), dried over $MgSO_4$, filtered and the solvent was removed under reduced pressure. The crude was purified by chromatography (EtOAc/cyclohexane 5→80%) to afford (3-bromo-5-(methylsulfinyl)phenyl)(methyl)sulfane **5** as an orange oil (6.26 g, 19% from **3**). **^{1}H NMR** (500 MHz, Chloroform-*d*) δ 7.46 – 7.41 (m, 3H), 2.75 (s, 3H), 2.53 (s, 3H). **^{13}C NMR** (126 MHz, Chloroform-*d*) δ 148.35, 143.41, 130.46, 123.61, 122.31, 118.88, 44.06, 15.34**. LCMS** (ESI) *m/z* 265/267 $[M+H]^+$

d) TFAA (6.30 mL, 43.3 mmol) was added to a solution of (3-bromo-5-(methylsulfinyl)phenyl)(methyl)sulfane **5** (4.0 g, 15.1 mmol) and 2,6-

lutidine (7.04 mL, 60.4 mmol) in MeCN (101 mL). The reaction was stirred at rt for 0.5 h and all volatiles were removed under reduced pressure. 1:1 Et_3N/MeOH (100 mL) was then added. The solution was stirred at rt for 0.5 h and was subsequently acidified to pH <4 with 2M HCl. The aqueous phase was extracted with EtOAc (400 mL). The organic phase was washed with 0.5 M HCl (200 mL) and brine (200 mL), dried over $MgSO_4$, filtered and the solvent was removed under reduced pressure. The crude was purified by chromatography (EtOAc/cyclohexane 0→30%) to afford 3-bromo-5-(methylthio)benzenethiol **6** as an orange oil (3.42 g, 96%). **^{1}H NMR** (500 MHz, Chloroform-*d*) δ 7.18 (m, 1H), 7.14 (m, 1H), 7.04 (m, 1H), 3.49 (s, 1H), 2.47 (s, 3H). **^{13}C NMR** (126 MHz, Chloroform-*d*) δ 141.86, 133.65, 127.99, 125.72, 124.83, 123.03, 15.49. **LCMS** – did not ionise.

*Synthetic route for compound **2***

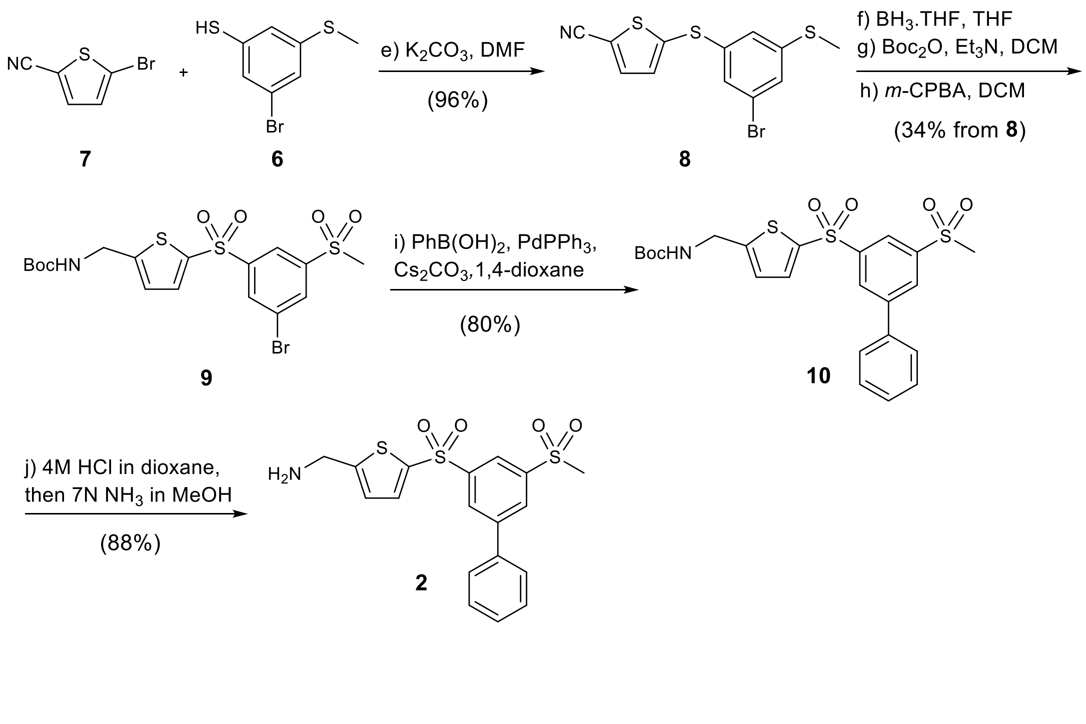

e) A mixture of 5-bromothiophene-2-carbonitrile **7** (5.20 g, 27.6 mmol), 3-bromo-5-(methylthio)benzenethiol **6** (5.41 g, 23.0 mmol), K_2CO_3

(4.77 g, 34.5 mmol) and DMF (115 mL) was stirred at 40 °C for 16 h. After cooling to rt, the mixture was diluted with EtOAc. The organic phase was washed with 1:1 H_2O/brine (3 ×), dried over $MgSO_4$ and filtered. The solvent was removed under reduced pressure. The crude was purified by chromatography (EtOAc/cyclohexane 0→20%) to afford 5-((3-bromo-5-(methylthio)phenyl)thio)thiophene-2-carbonitrile **8** as a yellow oil (7.53 g, 96%). **^{1}H NMR** (500 MHz, Chloroform-*d*) δ 7.57 (d, *J* = 3.9 Hz, 1H), 7.25 (t, *J* = 1.7 Hz, 1H), 7.21 (d, *J* = 3.8 Hz, 1H), 7.16 (t, *J* = 1.6 Hz, 1H), 7.09 (t, *J* = 1.6 Hz, 1H), 2.47 (s, 3H). **^{13}C NMR** (126 MHz, Chloroform-*d*) δ 142.81, 141.05, 138.03, 137.77, 134.07, 128.10, 127.78, 125.10, 123.63, 113.71, 113.35, 15.52. **LCMS** (ESI) *m/z* 342/344 $[M+H]^+$.

f) and **g**) BH_3.THF (1.0 M in THF; 66.1 mL, 66.1 mmol) was added to a solution of 5-((3-bromo-5-(methylthio)phenyl)thio)thiophene-2-carbonitrile **8** (7.53g, 22.0 mmol) in THF (66 mL) and the mixture was stirred at rt for 1 h. EtOH (132 mL) was carefully added to quench the reaction. Subsequently, the solution was heated at 70 °C for 1 h to aid decomplexation. The solvent was removed under reduced pressure. The crude was dissolved in DCM (110 mL). Et_3N (9.21 mL, 66.1 mmol), followed by Boc_2O (7.60 mL, 33.0 mmol) were added and the mixture was stirred at rt for 16 h. When complete conversion was achieved, more DCM was added (110 mL). The organic phase was washed with H_2O (220 mL) and brine (220 mL), dried over $MgSO_4$ and filtered. The solvent was removed under reduced pressure. The crude was used in the subsequent transformation immediately.

h) *m*-CPBA (50-55%; 32.6 g, ~99.1 mmol) was added in small portions to a solution of *tert*-butyl ((5-((3-bromo-5-(methylthio)phenyl)thio)thiophen-2-yl)methyl)carbamate (crude of step **f** and **g**) in DCM (148 mL) at 0 °C and the mixture was stirred at rt for 3 h. When complete conversion was achieved, EtOAc was added (250 mL). The organic phase was washed with sat. $NaHCO_3$ (4 × 200 mL) and sat. $Na_2S_2O_3$ (200 mL), dried over $MgSO_4$ and filtered. The

solvent was removed under reduced pressure. The crude was purified by column chromatography (EtOAc/cyclohexane 0→60%) to afford *tert*-butyl ((5-((3-bromo-5-(methylsulfonyl)phenyl)sulfonyl)thiophen-2-yl)methyl)carbamate as a yellow foam **9** (3.83 g, 34%). **^{1}H NMR** (500 MHz, Chloroform-*d*) δ 8.41 (m, 1H), 8.32 (m, 1H), 8.24 (m, 1H), 7.63 (d, *J* = 3.6 Hz, 1H), 6.97 (d, *J* = 3.9 Hz, 1H), 5.15 (br, 1H), 4.47 (m, 2H), 3.12 (s, 3H), 1.47 (s, 9H). **^{13}C NMR** (126 MHz, Chloroform-*d*) δ 155.07, 145.73, 143.77, 138.80, 135.21, 135.07, 134.81, 126.06, 124.89, 124.59, 80.68, 44.48, 39.91, 28.42. **LCMS** (ESI) *m/z* 532/534 $[M+Na]^+$, 393/395 $[M-BocNH]^+$.

i) A mixture of *tert*-butyl ((5-((3-bromo-5-(methylsulfonyl)phenyl)sulfonyl)thiophen-2-yl)methyl)carbamate **9** (2.00 g, 3.92 mmol), $Pd(PPh_3)_4$ (453 mg, 10%), benzeneboronic acid (574 mg, 4.71 mmol), Cs_2CO_3 (1.53 g, 4.71 mmol) and 1,4-dioxane (13 mL) was degassed with argon and then stirred at 100 ^{o}C for 16 h. After cooling to rt. The suspension was filtered through celite, washed with DCM and the solvent was removed under reduced pressure. The crude was purified by chromatography (EtOAc/cyclohexane 0→60%) to afford *tert*-butyl ((5-((5-(methylsulfonyl)-[1,1'-biphenyl]-3-yl)sulfonyl)thiophen-2-yl)methyl)carbamate **10** as a brown foam (1.59 g, 80%). **^{1}H NMR** (500 MHz, Chloroform-*d*) δ 8.47 (m, 1H), 8.42 (m, 1H), 8.33 (m, 1H), 7.70 – 7.62 (m, 3H), 7.58 – 7.43 (m, 3H), 6.96 (d, *J* = 3.8 Hz, 1H), 5.15 (br, 1H), 4.53 – 4.42 (m, 2H), 3.15 (s, 3H), 1.43 (s, 9H). **^{13}C NMR** (126 MHz, Chloroform-*d*) δ 154.41, 144.61, 144.58, 142.79, 139.48, 137.18, 134.67, 130.35, 130.09, 129.44, 127.34, 125.88, 124.56, 124.53, 80.38, 44.44, 39.77, 28.32**.** **LCMS** (ESI) *m/z* 530 $[M+Na]^+$, 391 $[M-BocNH]^+$.

j) 4M HCl in dioxane (10.5 mL) was added to a solution of *tert*-butyl ((5-((5-(methylsulfonyl)-[1,1'-biphenyl]-3-yl)sulfonyl)thiophen-2-yl)methyl)carbamate **10** (1.59 g, 3.14 mmol) in 1,4-dioxane (10.5 mL) and the mixture was stirred at rt 1or 16 h. Cyclohexane was added to precipitate the solid product. The solids were filtered, washed with

EtOAC/cyclohexane (1:1) and then treated with 2N NH_3 in MeOH. The solvent was removed under reduced pressure to afford compound **2** as a light brown solid (1.12 g, 88%). mp: 98-101 °C. ^{1}**H NMR** (500 MHz, Chloroform-*d*) δ 8.46 (t, *J* = 1.7 Hz, 1H), 8.44 (t, *J* = 1.7 Hz, 1H), 8.32 (t, *J* = 1.7 Hz, 1H), 7.67 (d, *J* = 3.9 Hz, 1H), 7.65 – 7.61 (m, 2H), 7.55 – 7.45 (m, 3H), 6.93 (m, 1H), 4.09 (s, 2H), 3.14 (s, 3H), 1.68 (br, 2H). ^{13}**C NMR** (126 MHz, Chloroform-*d*) δ 145.00, 144.60, 142.82, 138.62, 137.33, 134.96, 130.41, 130.02, 129.54, 127.42, 124.66, 124.18, 44.56, 41.68. **HRMS** (ESI) for $C_{18}H_{18}NO_4S_3$ ($[M+H]^+$): Calculated 408.0392; Observed 408.0401.

Spectra data for compound ***2*** *(CCT365623) and synthetic intermediates*

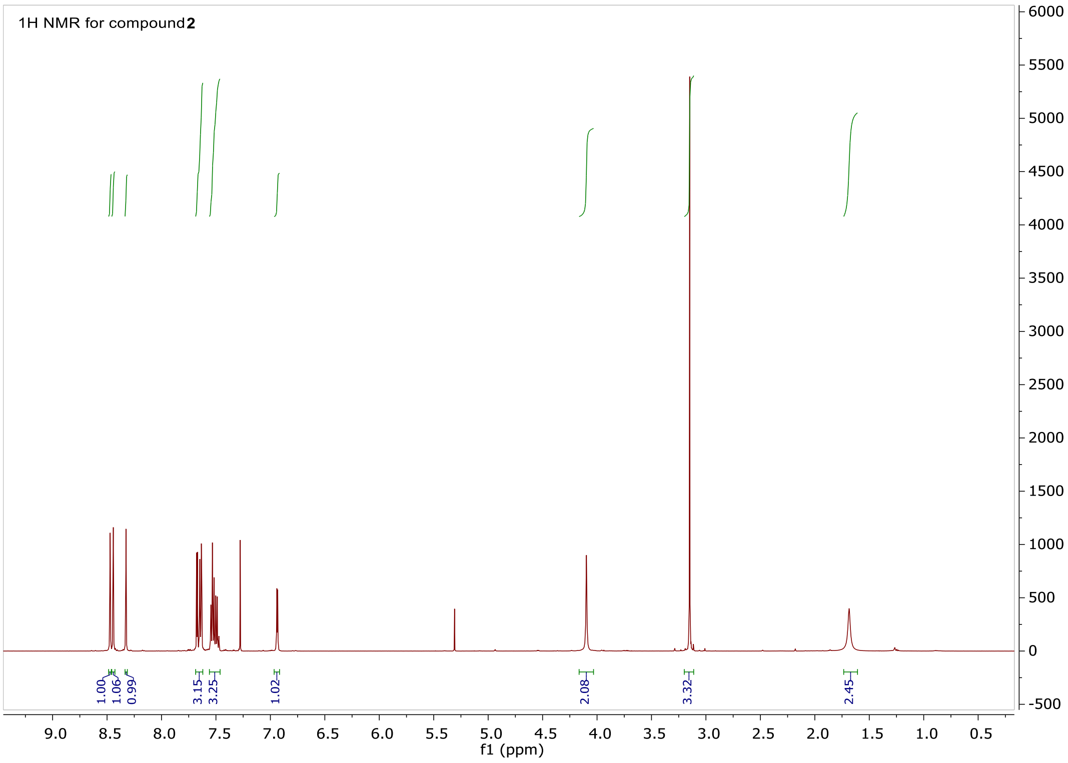

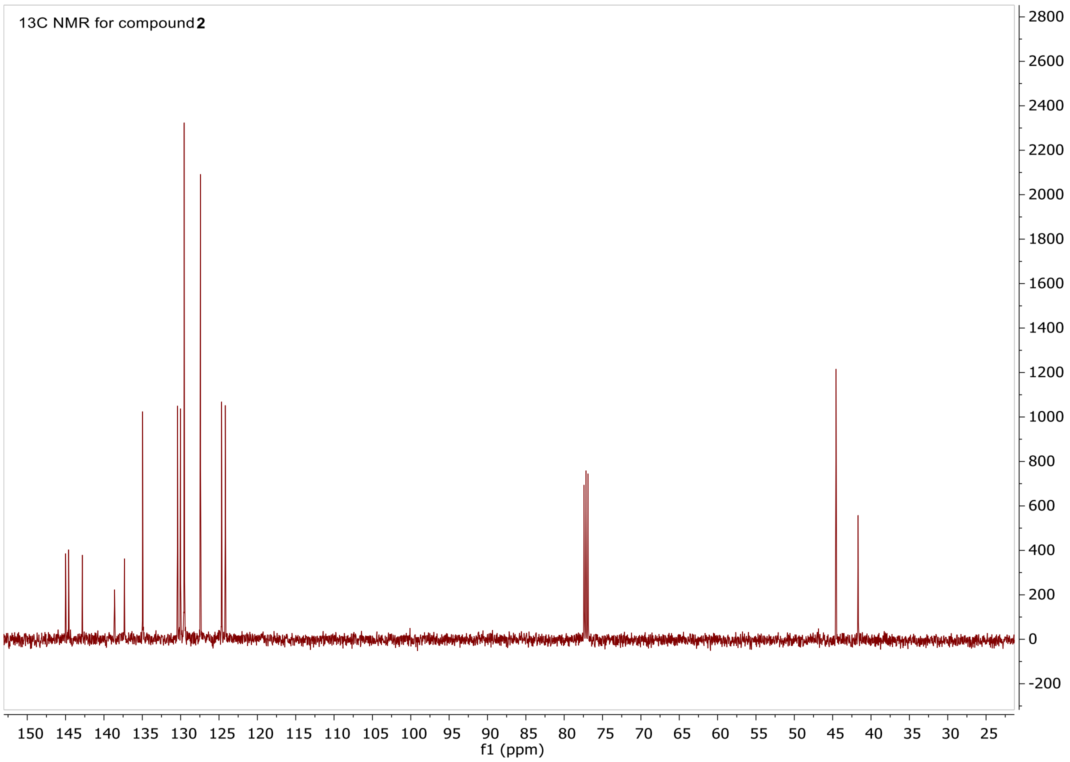
13C NMR for compound 2
2800
2600
2400
2200
2000
1800
1600
1400
1200
1000
800
600
400
200
0
-200
150 145 140 135 130 125 120 115 110 105 100 95 90 85 80 75 70 65 60 55 50 45 40 35 30 25
f1 (ppm)

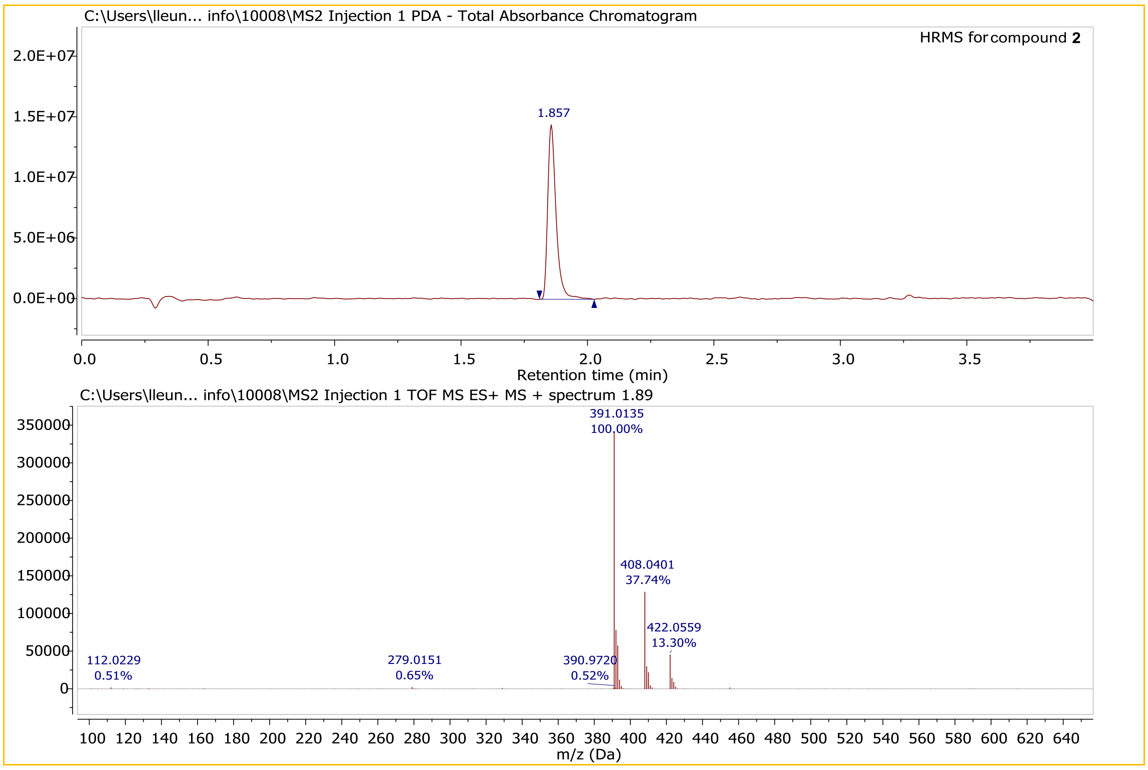
C:\Users\lleun... info\10008\MS2 Injection 1 PDA - Total Absorbance Chromatogram
HRMS for compound 2
2.0E+07
1.5E+07
1.0E+07
5.0E+06
0.0E+00
1.857
0.0 0.5 1.0 1.5 2.0 2.5 3.0 3.5
Retention time (min)
C:\Users\lleun... info\10008\MS2 Injection 1 TOF MS ES+ MS + spectrum 1.89
350000
300000
250000
200000
150000
100000
50000
0
391.0135
100.00%
408.0401
37.74%
422.0559
13.30%
112.0229
0.51%
279.0151
0.65%
390.9720
0.52%
100 120 140 160 180 200 220 240 260 280 300 320 340 360 380 400 420 440 460 480 500 520 540 560 580 600 620 640
m/z (Da)

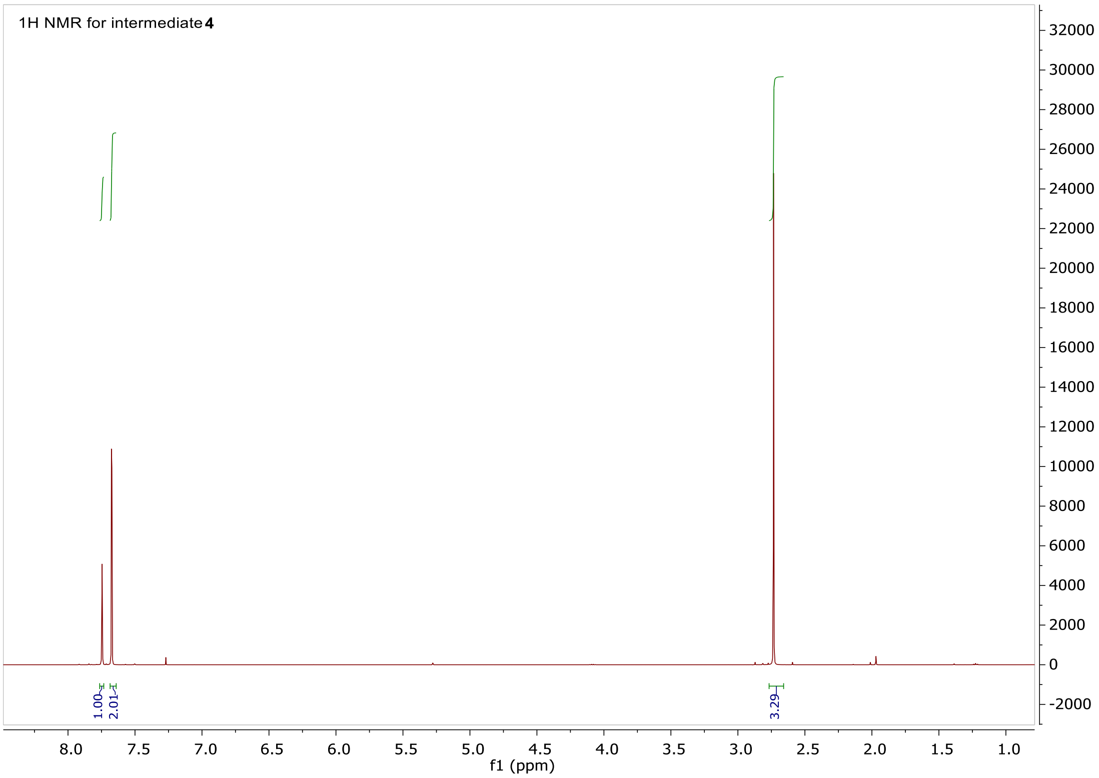

1H NMR for intermediate **4**
1.00
2.01
3.29
f1 (ppm)

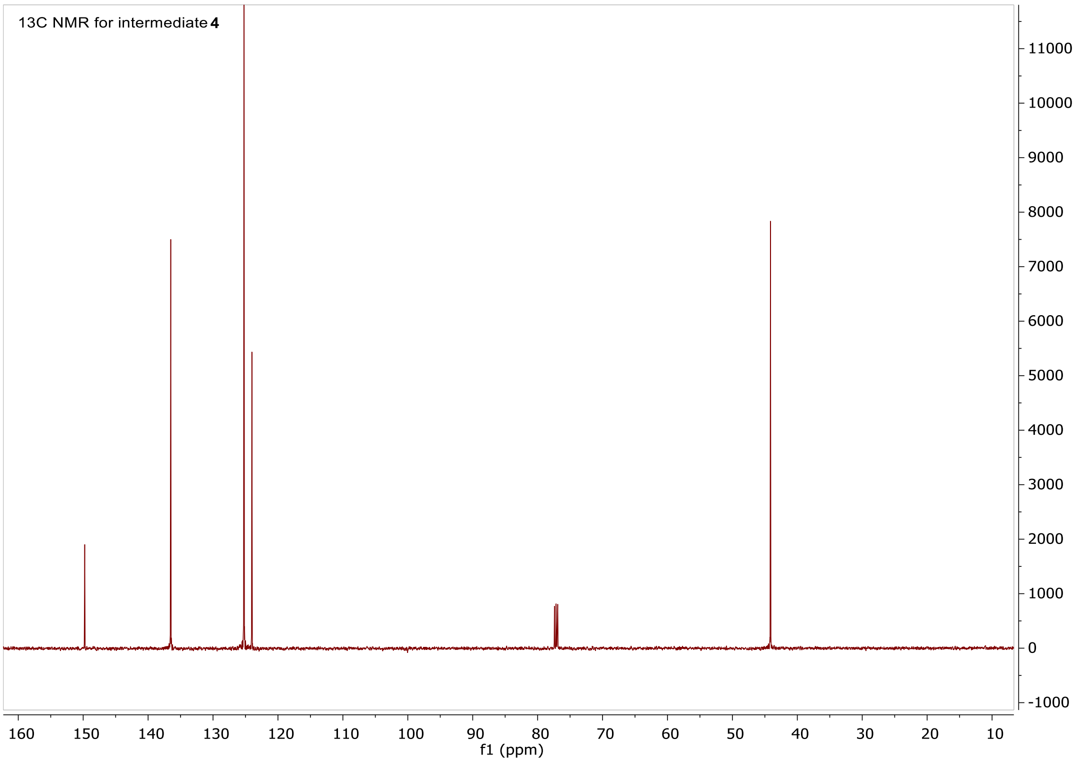

13C NMR for intermediate **4**
f1 (ppm)

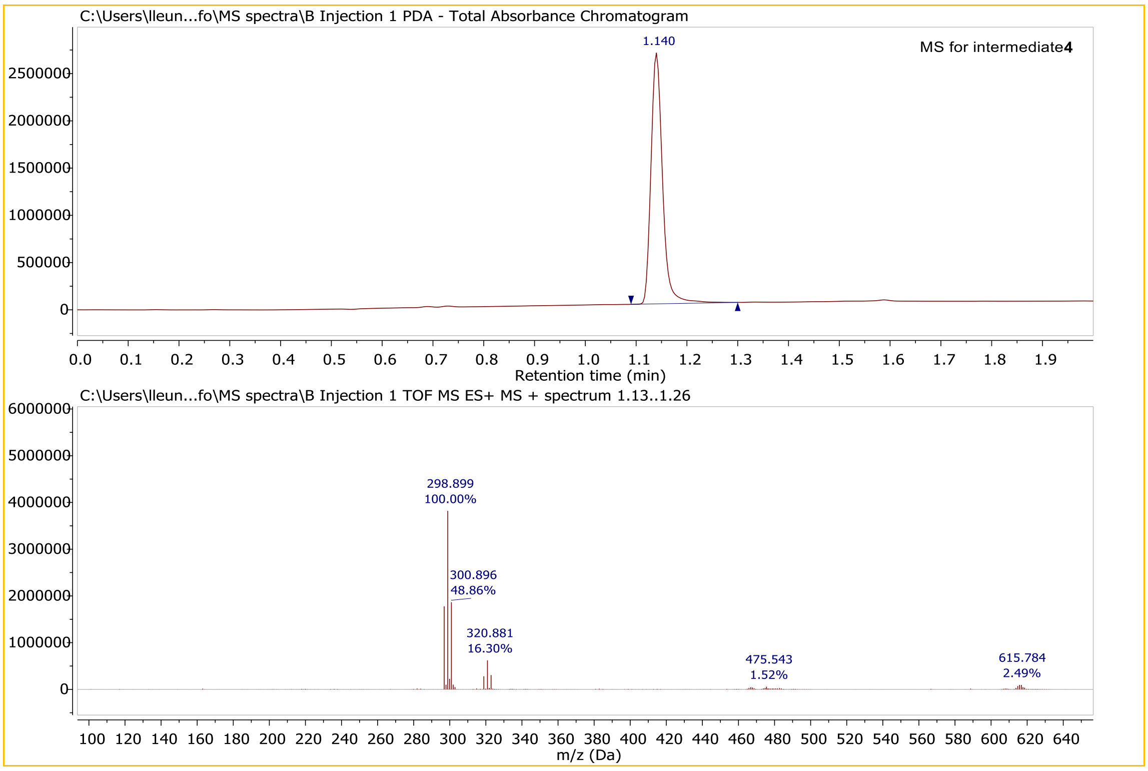
C:\Users\lleun...fo\MS spectra\B Injection 1 PDA - Total Absorbance Chromatogram
MS for intermediate **4**
1.140
Retention time (min)
C:\Users\lleun...fo\MS spectra\B Injection 1 TOF MS ES+ MS + spectrum 1.13..1.26
298.899
100.00%
300.896
48.86%
320.881
16.30%
475.543
1.52%
615.784
2.49%
m/z (Da)

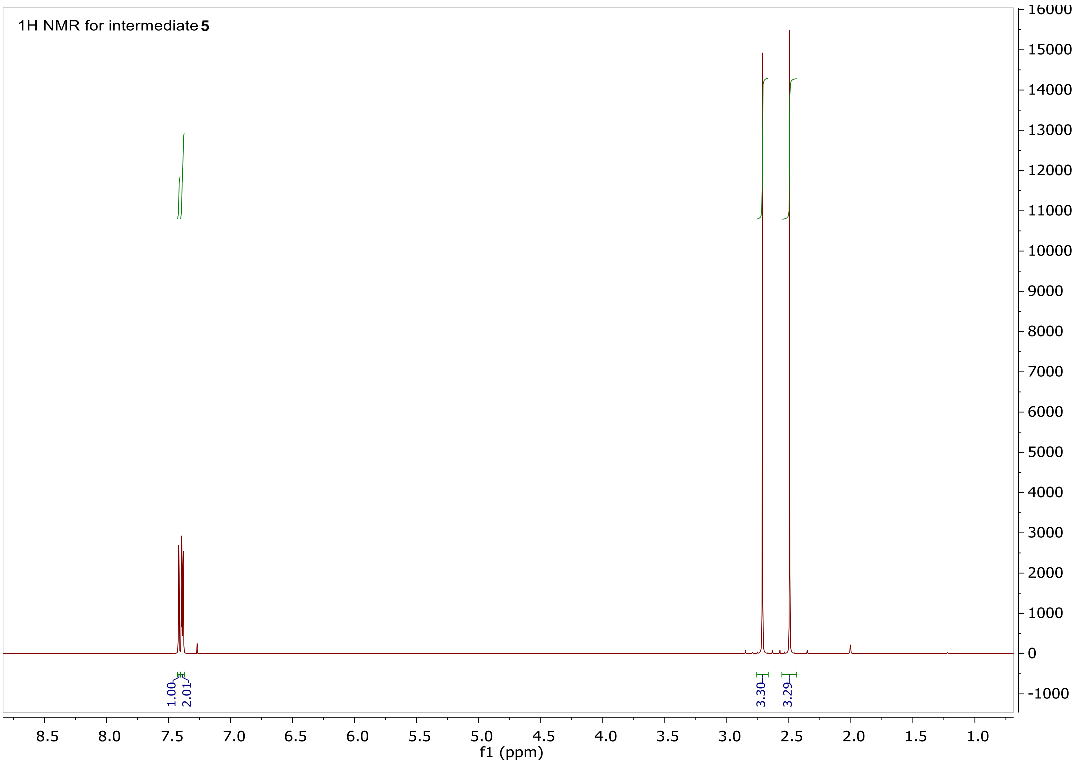
1H NMR for intermediate **5**
1.00
2.01
3.30
3.29
f1 (ppm)

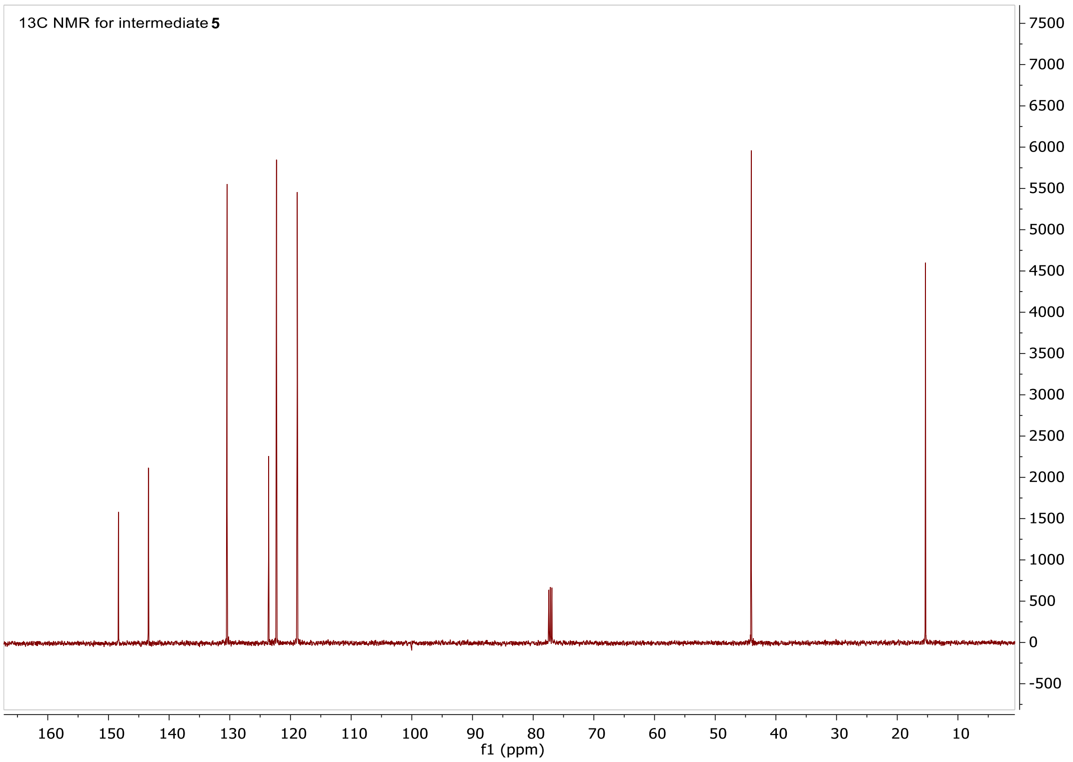
13C NMR for intermediate 5
7500
7000
6500
6000
5500
5000
4500
4000
3500
3000
2500
2000
1500
1000
500
0
-500
160 150 140 130 120 110 100 90 80 70 60 50 40 30 20 10
f1 (ppm)

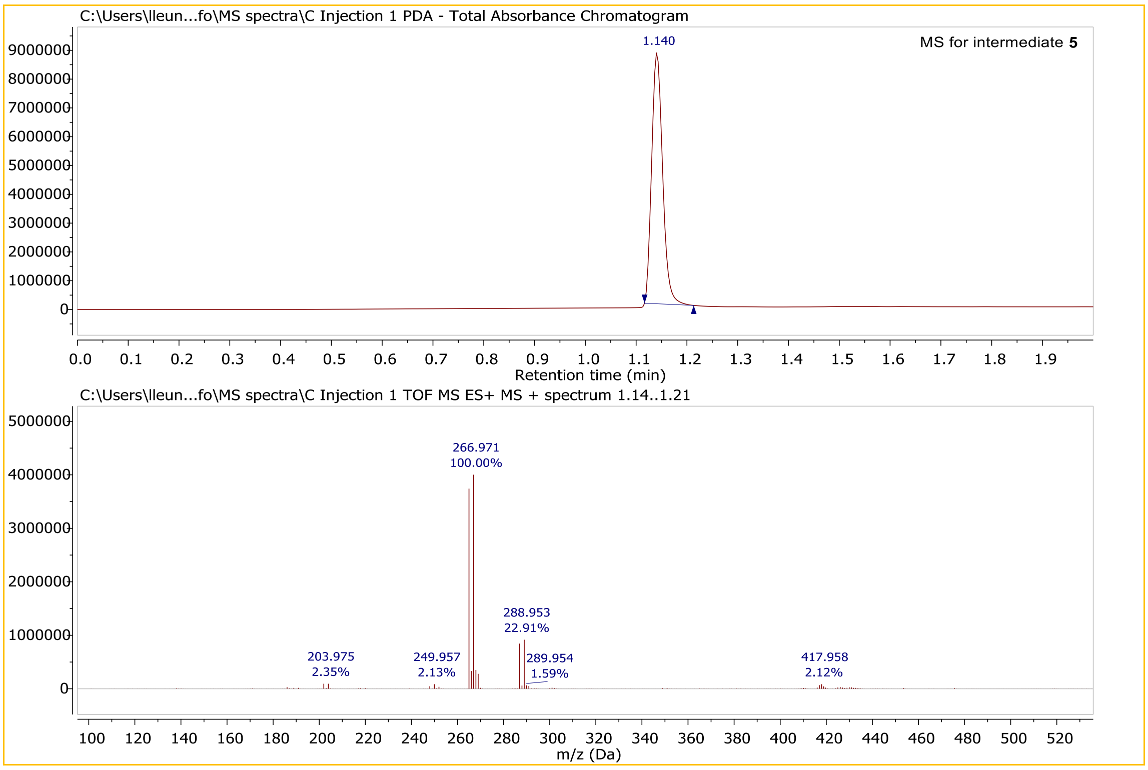
C:\Users\lleun...fo\MS spectra\C Injection 1 PDA - Total Absorbance Chromatogram
MS for intermediate 5
1.140
9000000
8000000
7000000
6000000
5000000
4000000
3000000
2000000
1000000
0
0.0 0.1 0.2 0.3 0.4 0.5 0.6 0.7 0.8 0.9 1.0 1.1 1.2 1.3 1.4 1.5 1.6 1.7 1.8 1.9
Retention time (min)
C:\Users\lleun...fo\MS spectra\C Injection 1 TOF MS ES+ MS + spectrum 1.14..1.21
5000000
4000000
3000000
2000000
1000000
0
266.971
100.00%
288.953
22.91%
203.975
2.35%
249.957
2.13%
289.954
1.59%
417.958
2.12%
100 120 140 160 180 200 220 240 260 280 300 320 340 360 380 400 420 440 460 480 500 520
m/z (Da)

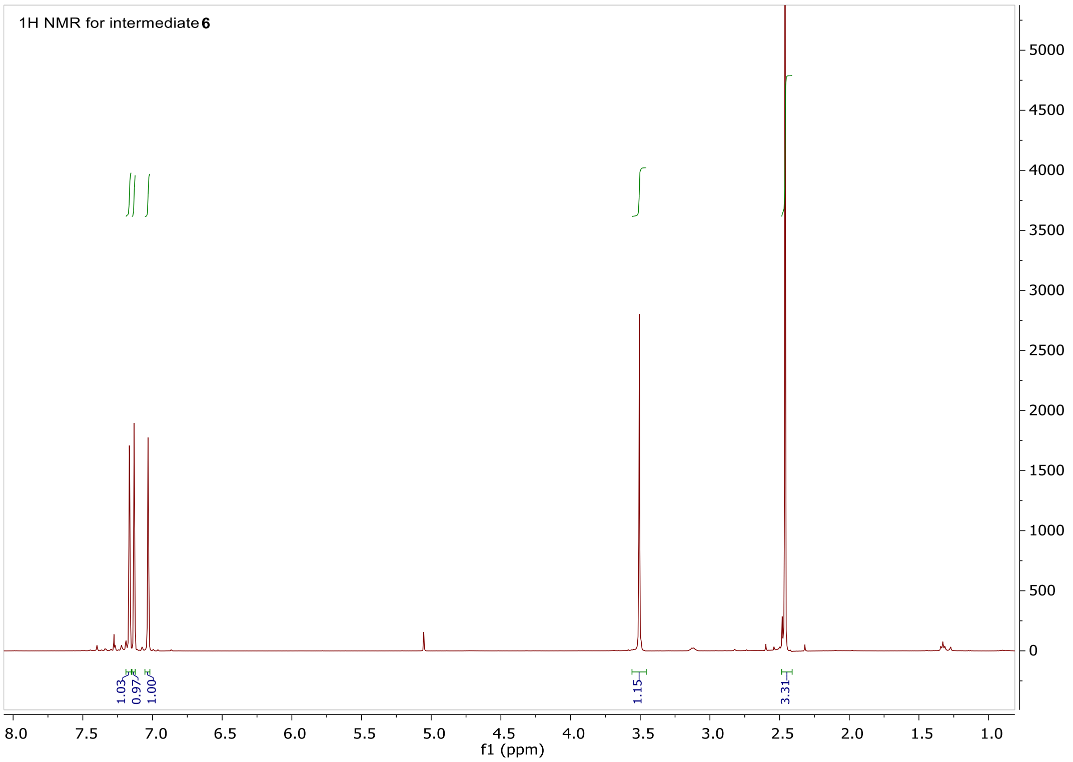

1H NMR for intermediate **6**
1.03
0.97
1.00
1.15
3.31
f1 (ppm)

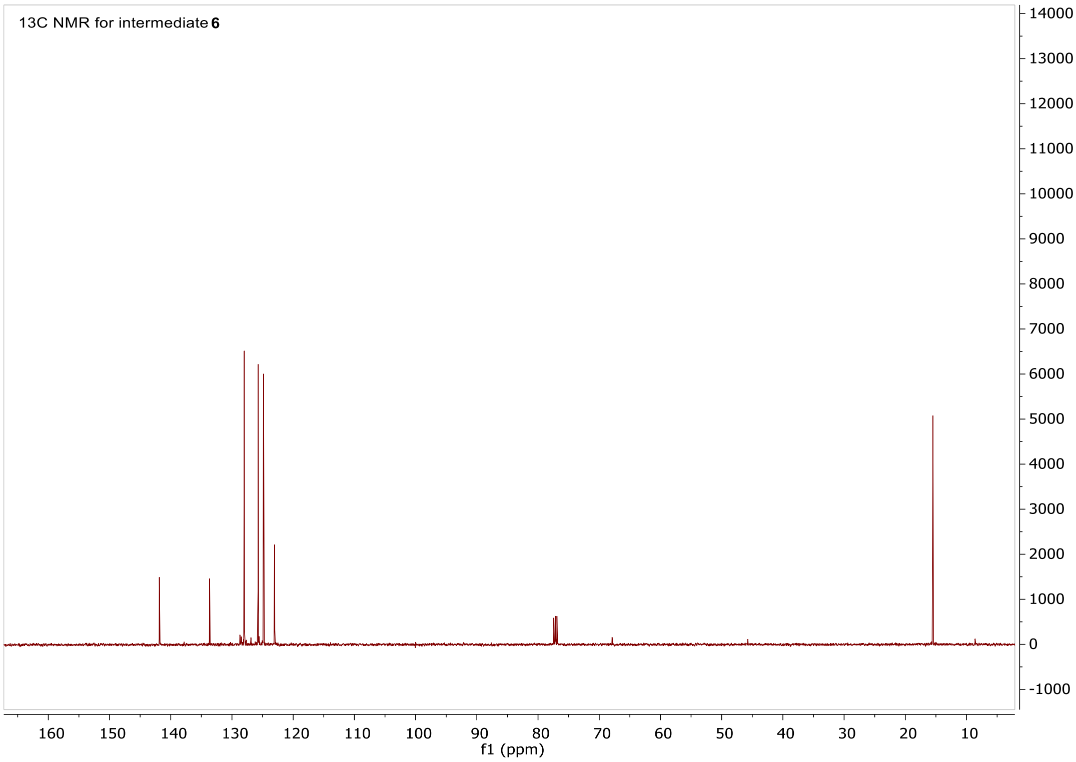

13C NMR for intermediate **6**
f1 (ppm)

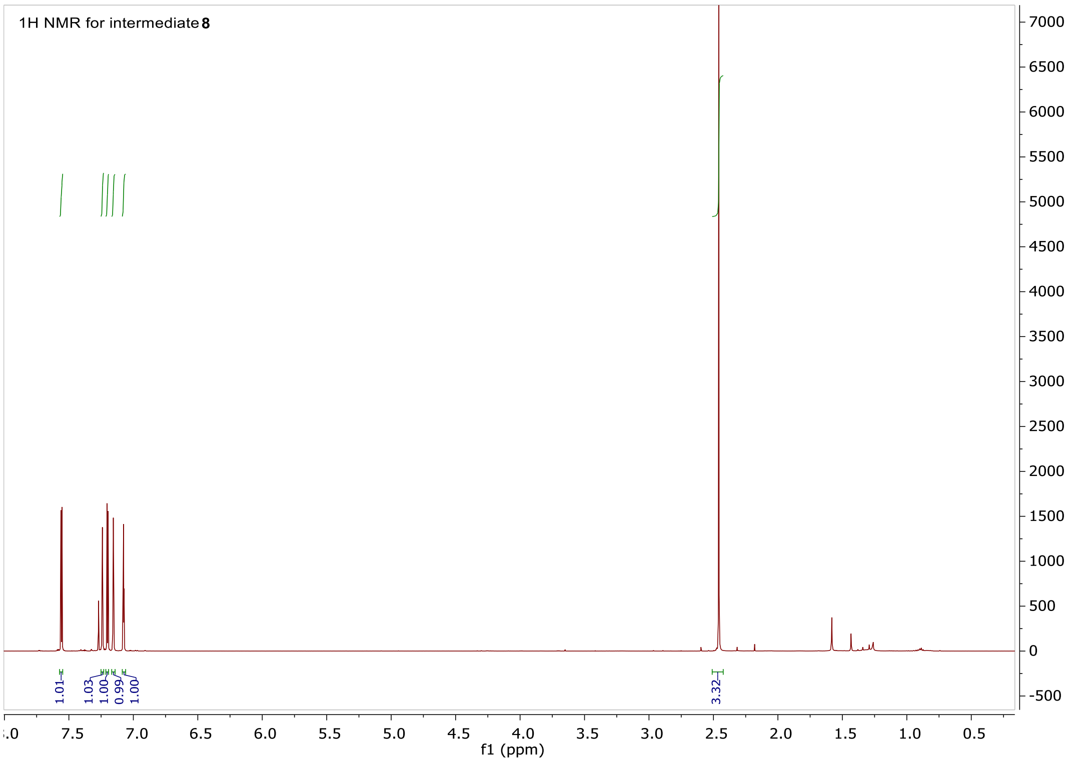
1H NMR for intermediate 8
1.01
1.03
1.00
0.99
1.00
3.32
f1 (ppm)

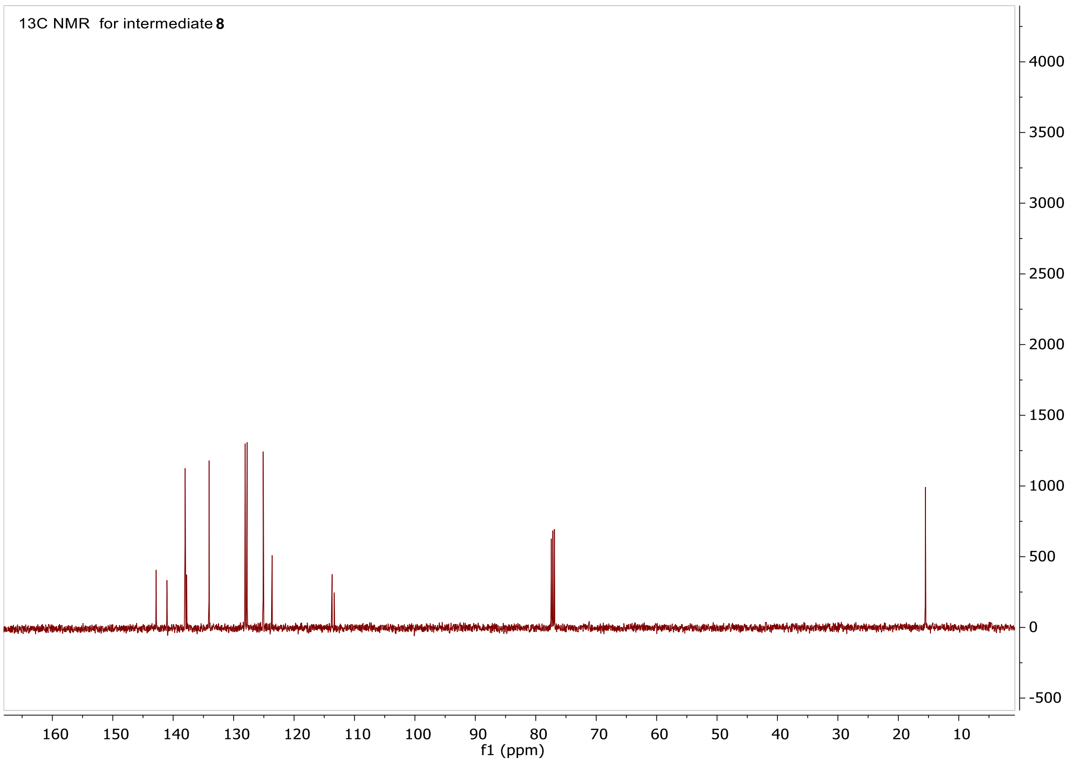
13C NMR for intermediate 8
f1 (ppm)

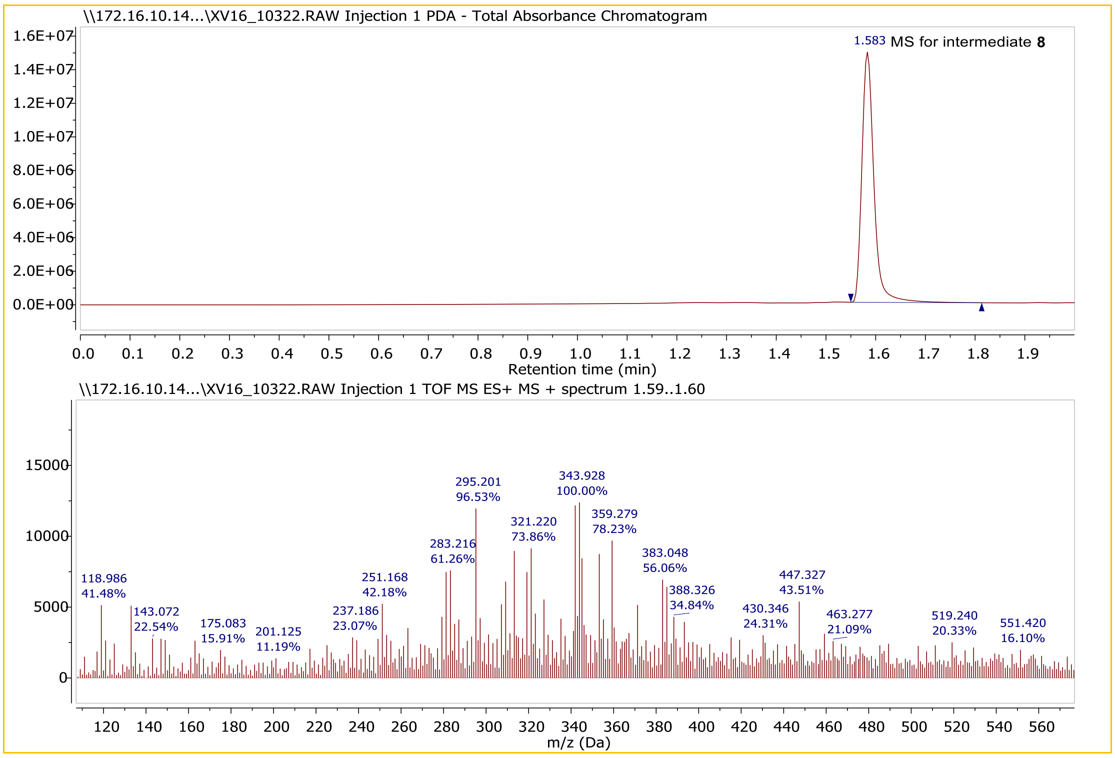
\\172.16.10.14...\XV16_10322.RAW Injection 1 PDA - Total Absorbance Chromatogram
1.583 MS for intermediate **8**
Retention time (min)
\\172.16.10.14...\XV16_10322.RAW Injection 1 TOF MS ES+ MS + spectrum 1.59..1.60
118.986 41.48%
143.072 22.54%
175.083 15.91%
201.125 11.19%
237.186 23.07%
251.168 42.18%
283.216 61.26%
295.201 96.53%
321.220 73.86%
343.928 100.00%
359.279 78.23%
383.048 56.06%
388.326 34.84%
430.346 24.31%
447.327 43.51%
463.277 21.09%
519.240 20.33%
551.420 16.10%
m/z (Da)

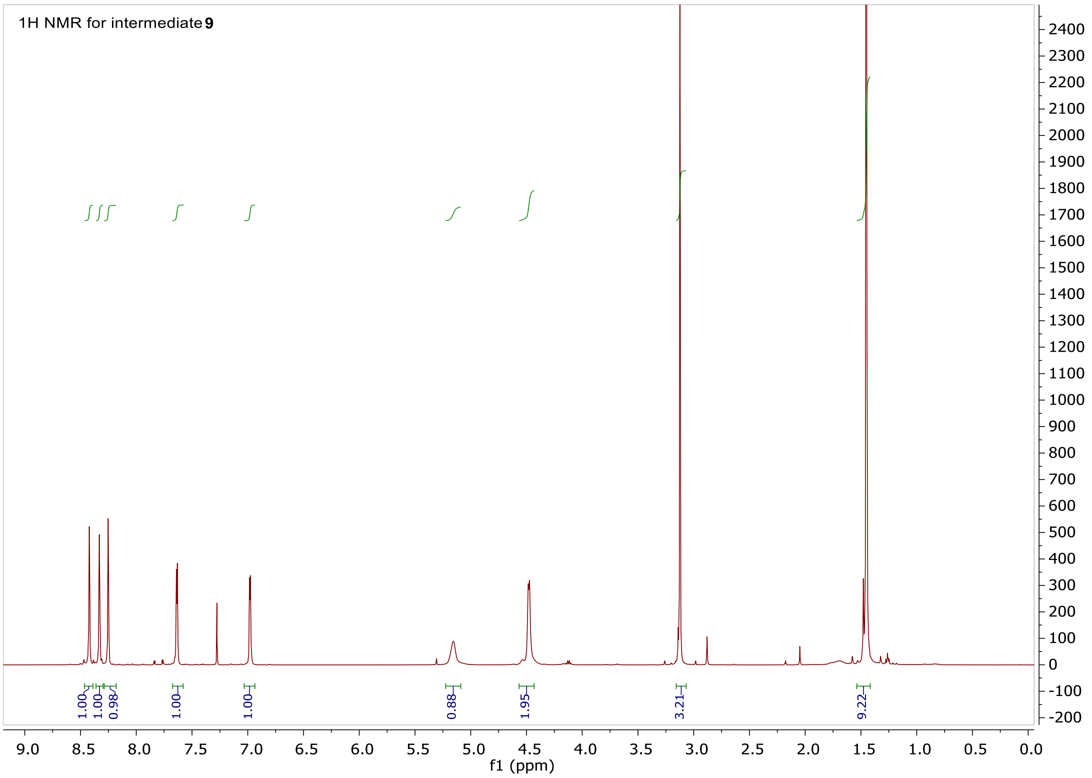
1H NMR for intermediate **9**
1.00
1.00
0.98
1.00
1.00
0.88
1.95
3.21
9.22
f1 (ppm)

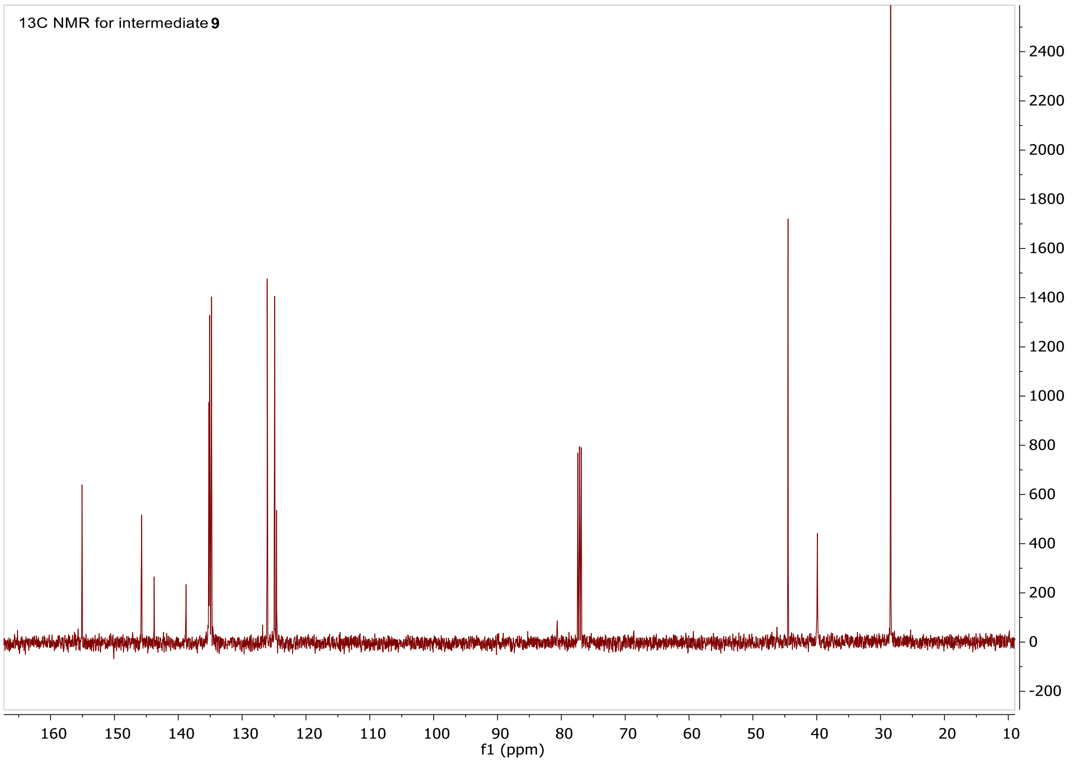

13C NMR for intermediate 9
f1 (ppm)

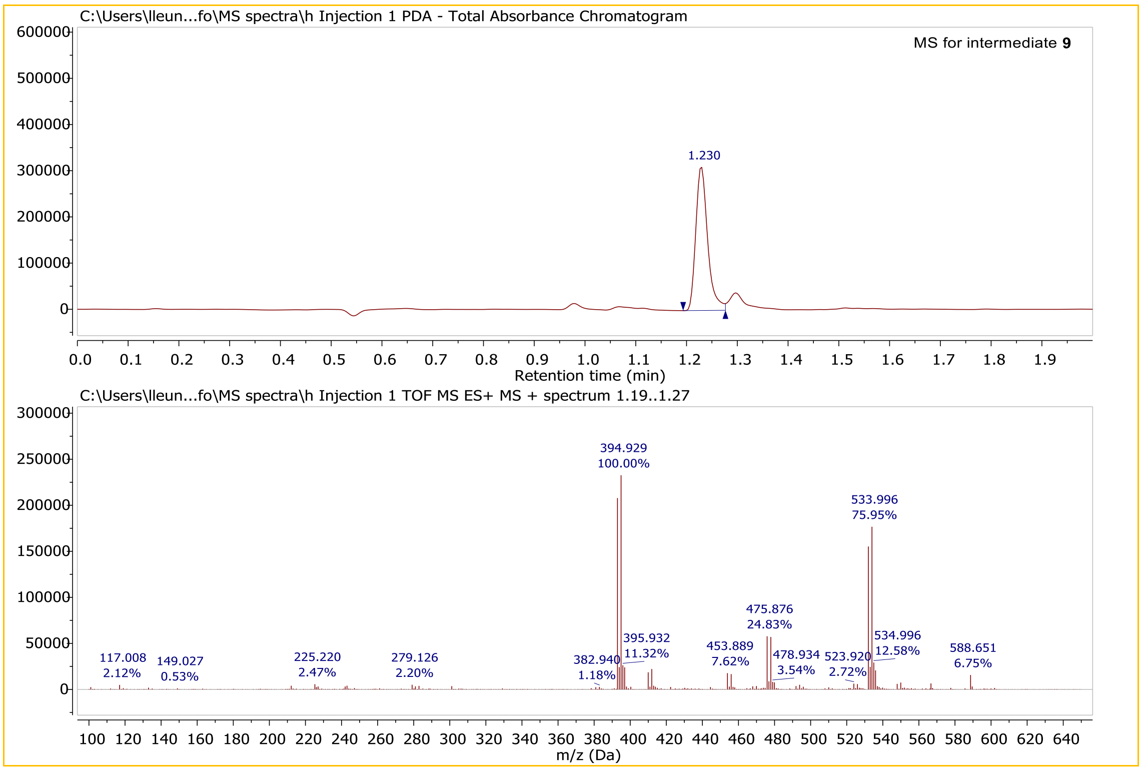

C:\Users\lleun...fo\MS spectra\h Injection 1 PDA - Total Absorbance Chromatogram
MS for intermediate 9
1.230
Retention time (min)
C:\Users\lleun...fo\MS spectra\h Injection 1 TOF MS ES+ MS + spectrum 1.19..1.27
394.929
100.00%
533.996
75.95%
475.876
24.83%
395.932
11.32%
453.889
7.62%
534.996
12.58%
588.651
6.75%
117.008
2.12%
149.027
0.53%
225.220
2.47%
279.126
2.20%
382.940
1.18%
478.934
3.54%
523.920
2.72%
m/z (Da)

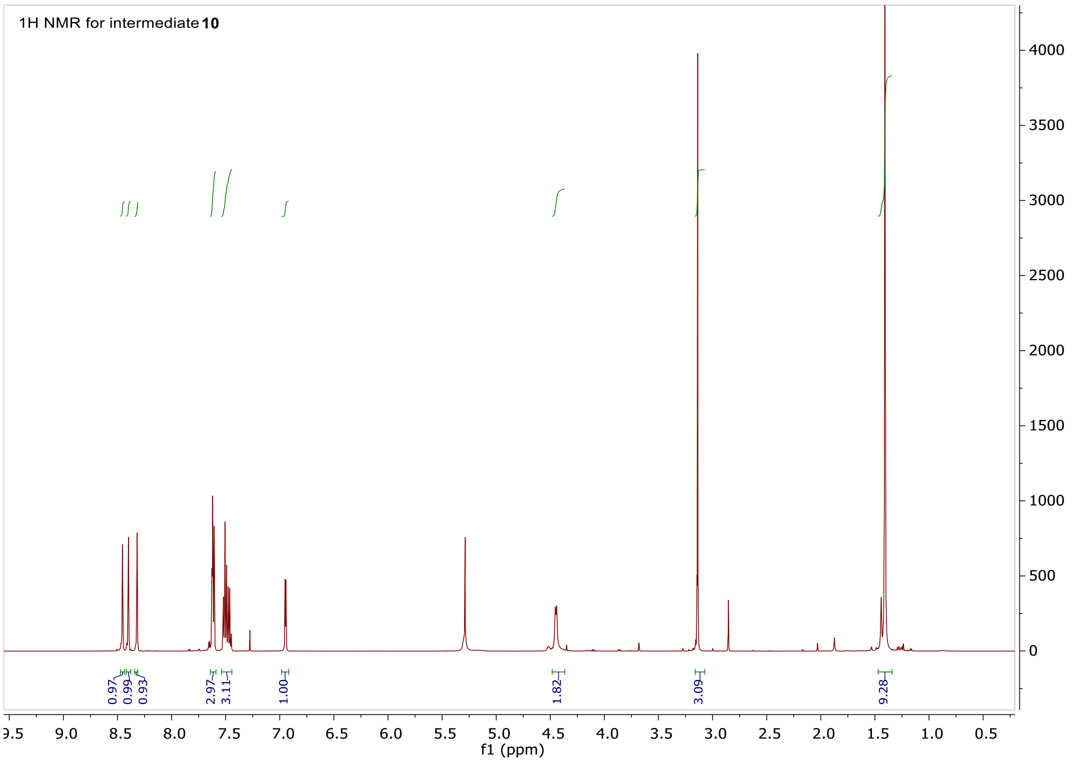
1H NMR for intermediate **10**
f1 (ppm)

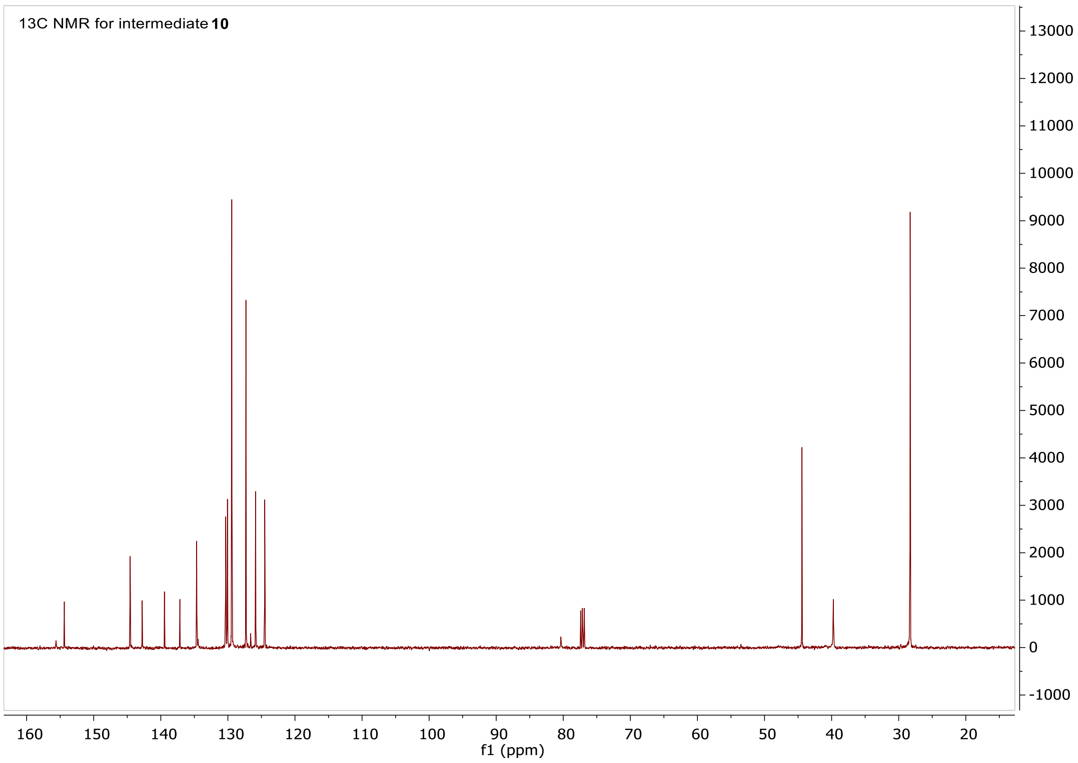
13C NMR for intermediate **10**
f1 (ppm)

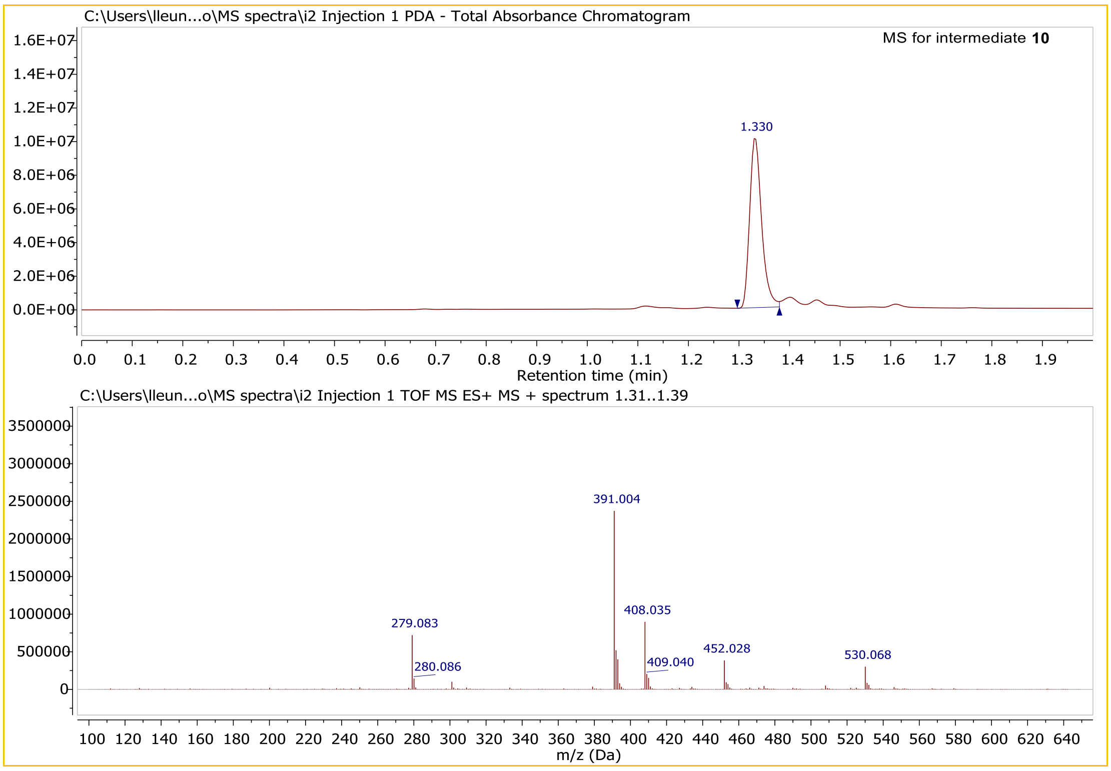
C:\Users\lleun...o\MS spectra\i2 Injection 1 PDA - Total Absorbance Chromatogram
MS for intermediate 10
1.330
Retention time (min)
C:\Users\lleun...o\MS spectra\i2 Injection 1 TOF MS ES+ MS + spectrum 1.31..1.39
391.004
408.035
279.083
280.086
409.040
452.028
530.068
m/z (Da)
